# Log Transformation Improves Dating of Phylogenies

**DOI:** 10.1093/molbev/msaa222

**Published:** 2020-09-04

**Authors:** Uyen Mai, Siavash Mirarab

**Affiliations:** 1 Department of Computer Science and Engineering, UC, San Diego, CA; 2 Department of Electrical and Computer Engineering, UC, San Diego, CA

**Keywords:** time tree, divergence time estimation, phylogenetic dating, molecular dating, nonconvex optimization

## Abstract

Phylogenetic trees inferred from sequence data often have branch lengths measured in the expected number of substitutions and therefore, do not have divergence times estimated. These trees give an incomplete view of evolutionary histories since many applications of phylogenies require time trees. Many methods have been developed to convert the inferred branch lengths from substitution unit to time unit using calibration points, but none is universally accepted as they are challenged in both scalability and accuracy under complex models. Here, we introduce a new method that formulates dating as a nonconvex optimization problem where the variance of log-transformed rate multipliers is minimized across the tree. On simulated and real data, we show that our method, wLogDate, is often more accurate than alternatives and is more robust to various model assumptions.

## Introduction

Phylogenetic inference from sequence data does not reveal divergence time (i.e., exact timing of evolutionary events) unless paired with external timing information. Under standard models of sequence evolution, the evolutionary processes, including sequence divergence, are fully determined by the product of the absolute time and mutation rates in a nonidentifiable form. Thus, these models measure branch lengths in the unit of expected numbers of mutations per site (since standard models like GTR [Tavaré 1986] only allow substitutions, focusing on these models, we use *substitutions* and *mutations* interchangeably throughout this paper). Nevertheless, knowing divergence times is crucial for understanding evolutionary processes (Hillis et al. 1996; Forest 2009) and is a fundamental need in many clinical applications of phylogenetics and phylodynamics (Volz et al. 2013). A commonly used approach first infers a phylogeny with branch lengths in the unit of substitution per site and then dates the phylogeny by translating branch lengths from substitution unit to time unit; coestimation of topology and dates is also possible (Drummond et al. 2006) though its merits have been debated (Wertheim et al. 2010).

The fundamental challenge in dating is to find a way to factorize the number of substitutions into the product of the evolutionary rate and time. A common mechanism allowing this translation is to impose soft or hard constraints on the timing of *some* nodes of the tree, leaving the divergence times of the remaining nodes to be inferred based on the constrained nodes. Timing information is often in one of two forms: calibration points obtained from the geological record (Kodandaramaiah 2011) and imposed on either internal nodes or tips that represent fossils (see Donoghue and Yang 2016), or tip sampling times for fast-evolving viruses and bacteria. The constraints still leave us with a need to extrapolate from observed times for a few nodes to the remaining nodes, a challenging task that requires a mathematical approach. Obtaining accurate timing information and formulating the right method of extrapolation are both challenging (see Rutschmann 2006).

Many computational methods for dating phylogenies are available (see Rutschmann 2006; Kumar and Hedges 2016), and a main point of differentiation between these methods is the clock model they assume (Sanderson 1998). Some methods rely on a strict molecular clock (Zuckerkandl 1962) where rates are effectively assumed to be constant (Langley and Fitch 1974; Shankarappa et al. 1999). However, empirical evidence has now made it clear that rates can vary substantially, and ignoring these changes can lead to incorrect dating (Bromham and Penny 2003; Kumar 2005). Consequently, there have been many attempts to *relax* the molecular clock and allow variations in rates. A main challenge in relaxing the clock is the need for a model of rates, and it is not clear what model should be preferred. As a result, many methods for dating using relaxed molecular clocks have been developed. Some of these methods allow rates to be drawn independently from a stationary distribution (Drummond et al. 2006; Akerborg et al. 2008; Volz and Frost 2017) whereas others model the evolution of rates with time (Huelsenbeck et al. 2000) or allow correlated rates across branches (Thorne et al. 1998; Kishino et al. 2001; Sanderson 2002; Lepage et al. 2007; Drummond and Suchard 2010; Snir et al. 2012; Tamura et al. 2012). Despite these developments, strict molecular clocks continue to be used, especially in the context of intraspecific evolution where there is an expectation of relatively uniform rates (Brown and Yang 2011).

Another distinction between methods is the use of explicit models (Sanderson 1997). Many dating methods use a parametric statistical model and formulate dating as estimating parameters in a maximum likelihood (ML) or Bayesian inference framework (Langley and Fitch 1974; Drummond et al. 2006; To et al. 2016; Volz and Frost 2017). Another family of methods (Sanderson 2002; Tamura et al. 2012) formulate dating as optimization problems, including distance-based optimization (Xia and Yang 2011; Xia 2018), that avoid computing likelihood under an explicit statistical model. When the assumed parametric model is close to the reality, we expect parametric methods to perform well. However, these methods can be sensitive to model deviations, a problem that may be sidestepped by methods that avoid using specific models.

In this paper, we introduce (w)LogDate, a new method of dating rooted phylogenies that allows variations in rates but without modeling rates using specific distributions. We define mutation rates necessary to compute time unit branch lengths as the product of a single global rate and a set of rate multipliers, one per branch. We seek to find the overall rate and all rate multipliers such that the log-transformed rate multipliers have the minimum variance. This formulation gives us a constrained optimization problem, which although is not convex, can be solved in a scalable fashion using the standard approaches such as sequential least squares programing. While formulation of dating as an optimization problem is not new (Langley and Fitch 1974; To et al. 2016), here we introduce log-transformation of the rate multipliers, which as we will show, results in more accurate dates. Our observation is in line with a recent change to RelTime (Tamura et al. 2018) where the switch from arithmetic means to geometric means (between rates of sister lineages) has improved accuracy. In extensive simulation studies and three biological data sets, we show that a weighted version of LogDate, namely wLogDate, has higher accuracy in inferring node ages compared with alternative methods, including some that rely on time-consuming Bayesian inference. While wLogDate can date trees using both sampling times for leaves (e.g., in viral evolution) or estimated time of ancestors, most of our results are focused on cases with sampling times at the tips of the tree.

## Materials and Methods

### Definitions and Notations

For a rooted binary tree *T* with *n* leaves, we give each node a unique index in [0,…,2n−2]. By convention, the root is always assigned 0, the other internal nodes are arbitrarily assigned indices in the range [1,…,n−2], and the leaves are arbitrarily assigned indices in the range [n−1,…,2n−2]. In the rest of this paper, we will refer to any node by its index. If a node *i* is not the root node, we let par(*i*) denote the parent of *i* and if *i* is not a leaf, we let $c_{l}(i)$ and $c_{r}(i)$ denote the left and right children of *i*, respectively. We refer to the edge connecting par(*i*) and *i* as *e_i_*.

We can measure each edge *e_i_* of *T* in either time unit or substitution unit. Let *t_i_* denote the divergence time of node *i*, that is the time when species *i* diverged into $c_{l}(i)$ and $c_{r}(i)$. Then for any node *i* other than the root, $\tau_{i}=t_{i}-t_{\text{par}(i)}$ is the length of the edge *e_i_* in time unit. We measure divergence time of a node with respect to a fixed reference point in the past (i.e., time increases forward). Thus, we enforce $t_{i}>t_{\text{par}(i)}$ for all *i*. Let *μ_i_* be the substitution rate (per sequence site per time unit) on branch *e_i_*; then, the expected number of substitutions per sequence site is $b_{i}=\mu_{i}\tau_{i}$. Let $\tau =[\tau_{1},\ldots ,\tau_{2n-2}]$ and $b=[b_{1},\ldots ,b_{2n-2}]$.

From sequence data, b can be inferred using standard methods such as maximum parsimony (Fitch 1971), minimum evolution (Rzhetsky and Nei 1993), neighbor-joining (Saitou and Nei 1987; Gascuel 1997), and ML (Felsenstein 1981; Guindon et al. 2010; Nguyen et al. 2015). Note that inferred trees need to be rooted subsequently using an outgroup (that can be removed) or automatic methods such as midpoint or minimum variance rooting (Mai et al. 2017). We let ${\hat{b}}_{i}$ denote the estimate of *b_i_* by an inference method and let $\hat{b}=[{\hat{b}}_{1},\ldots ,{\hat{b}}_{2n-1}]$.

In this paper, we are interested in computing τ from $\hat{b}$. The computation of τ from $\hat{b}$ is complicated by two factors: 1) the possibility of change among rates, and 2) deviations of the inferred edge length ${\hat{b}}_{i}$ from the true value *b_i_*. To better describe the mathematical formulation of the optimization problem, we first do the following change of variables. Assuming the mutation rates on the branches are distributed around a global rate *μ*, we define $\nu_{i}=\mu \tau_{i}/{\hat{b}}_{i}$. Let $x=[\nu_{1},\ldots ,\nu_{2n-2},\mu ]$; our goal of finding τ is identical to finding x.

### Dating as a Constrained Optimization Problem

We formulate dating as an optimization problem on 2n−1 variables $x=[\nu_{1},\ldots ,\nu_{2n-2},\mu ]$, subject to the linear constraints defined by calibration points and/or sampling times. Many existing methods, including LF (Langley and Fitch 1974) and LSD (To et al. 2016), can be described in this framework, with the choice of the objective function distinguishing them from each other. We start by describing the setup of the constraints enforced by a set of calibration points/sampling times, and show that they can all be written as linear equations on x. We then give the formulation of both LF and LSD in this framework and use their formulation to motivate our own new approach. Finally, we describe strategies to solve the wLogDate optimization problem.

#### Linear Constraints Ψ from Sampling Times

For any pair of nodes (*i*, *j*) (where each of *i* and *j* can either be a leaf or an internal node) with enforced divergence times (*t_i_*, *t_j_*), the following constraint ψ(i,j) must be satisfied

(1) $$\psi (i,j):\mu (t_{j}-t_{i})=\sum_{k\in P(m,j)}\nu_{k}{\hat{b}}_{k}-\sum_{k\in P(i,m)}\nu_{k}{\hat{b}}_{k},$$

where *m* is the LCA of *i* and *j* and *P*(*m*, *j*) and *P*(*i*, *m*) are the paths connecting *m* to *j* and *i* to *m*, respectively. Thus, given *k* time points, k(k−1)/2 constraints must hold. However, only *k* − 1 of these constraints imply all others, as we show below.

Let *t*_0_ be the *unknown* divergence time at the root of the tree. For *k* calibration points $t_{1},\ldots ,t_{k}$, we can setup *k* constraints of the form:

(2) $$C_{i}:\mu (t_{i}-t_{0})=\sum_{k\in P(0,i)}\nu_{k}{\hat{b}}_{k},$$

where node 0 is the root and *P*(0, *i*) is the path from the root to node *i*. For any pair (*i*, *j*), the linear constraint given in equation (1) can be derived by subtracting *C_i_* from *C_j_* side by side. Also, we can remove *t*_0_ from the set of constraints by subtracting *C*_1_ from all other constraints $C_{2},\ldots ,C_{k}$. This gives us the *k* − 1 linear constraints on x, which we denote as Ψ. We can build Ψ using supplementary algorithm 1, Supplementary Material online.

#### Optimization Criteria

Since $\nu_{i}=\mu \tau_{i}/{\hat{b}}_{i}$, the distribution of *ν_i_* is influenced by both the distribution of the rates (*μ_i_*) and the distribution of ${\hat{b}}_{i}$ around *b_i_*. In traditional strict-clock models (Zuckerkandl 1962), a constant rate is assumed throughout the tree ($\forall_{i}\mu_{i}=\mu$). Under this model, the distribution of *ν_i_* is determined by deviations of ${\hat{b}}_{i}$ from *b_i_*.

Langley and Fitch (1974) (LF) modeled the number of *observed* substitutions *per sequence site* on a branch *i* by a Poisson distribution with mean $\lambda =\mu \tau_{i}$ and treated $s{\hat{b}}_{i}$ as if they were the total number of *observed* substitutions; as such, they assume $s{\hat{b}}_{i}∼\text{Poisson}(s\mu \tau_{i})$, where s is the sequence length. Therefore, by changing variable, we can write the log-likelihood function as:

$$\sum_{i=1}^{2n-2}\left(s{\hat{b}}_{i}\;\text{log}(s{\hat{b}}_{i})-\text{log}((s{\hat{b}}_{i})!)\right)+\sum_{i=1}^{2n-2}s{\hat{b}}_{i}\left(\text{log}\;\nu_{i}-\nu_{i}\right).$$

Given s and ${\hat{b}}_{i}$, LF finds x that maximizes the log-likelihood function and subject to the constraints Ψ. As such,

(3) $$\begin{array}{cc} x_{P}^{*}= & \underset{x}{\text{argmin}}\sum_{i=1}^{2n-2}{\hat{b}}_{i}(\nu_{i}-\text{log}\;\nu_{i})\;\text{subject to }\Psi . \end{array}$$

To et al. (2016) assume ${\hat{b}}_{i}$ follows a Gaussian model: ${\hat{b}}_{i}∼\text{Gaussian}(\mu \tau_{i},\sigma_{i}^{2})$ and approximate the variance by ${\hat{b}}_{i}/s$ (the method includes smoothing strategies omitted here). Then, the negative log-likelihood function can be written as:

$$\sum_{i=1}^{2n-2}\frac{{\left({\hat{b}}_{i}-\mu \tau_{i}\right)}^{2}}{\sigma_{i}^{2}}\approx \sum_{i=1}^{2n-2}\frac{s}{{\hat{b}}_{i}}{\left({\hat{b}}_{i}-\mu \tau_{i}\right)}^{2}=\sum_{i=1}^{2n-2}s{\hat{b}}_{i}{\left(1-\nu_{i}\right)}^{2}.$$

Thus, the ML estimate can be formulated as:

(4) $$\begin{array}{cc} x_{G}^{*}= & \underset{x}{\arg\min}\sum_{i=1}^{2n-2}{\hat{b}}_{i}{\left(1-\nu_{i}\right)}^{2}\;\;\text{subject}\;\text{to}\Psi . \end{array}$$

Both LF and LSD have convex formulations. Langley and Fitch (1974) proved that their negative log-likelihood function is convex and thus the local minimum is also the global minimum. Our constraint-based formulation of LF also can be easily proved convex by showing its Hessian matrix is positive definite. To et al. (2016) pointed out their objective function is a weighted least squares. Using our formulation, we also see that equation (4) together with the calibration constraints form a standard convex quadratic optimization problem which has a unique analytical solution.

### LogDate Method

#### Motivation

LF only seeks to model the errors in $\hat{b}$ and ignore true rate heterogeneity. Strict-clock assumption is now believed to be unrealistic in many settings (Schwartz and Maresca 2006; Pulquério and Nichols 2007; Ho 2014), motivating relaxed clocks, typically by assuming that *μ_i_*s are drawn i.i.d. from some distribution (Drummond et al. 2006; Akerborg et al. 2008; Volz and Frost 2017). Most methods rely on presumed parametric distributions (typically, LogNormal, Exponential, or Gamma) and estimate parameters using ML (Volz and Frost 2017), MAP (Akerborg et al. 2008), or MCMC (Drummond et al. 2006; Drummond and Rambaut 2007). The LSD method, which like LF directly models errors in $\hat{b}$, is additionally justified under a Gaussian clock model. Choices of specific distributions in these methods are not motivated by the knowledge that real data follow them exactly (for example, the Normal distribution has to be misspecified as mutation rates cannot be negative).

Our goal is to avoid explicit parameter inference under a model of rate multipliers. Instead, we follow the assumption shared by existing methods like LSD and LF: we assume that given two solutions of x both satisfying the calibration constraints, the solution with less variability in *ν_i_* values is preferable. Thus, we prefer solutions that minimize deviations from a strict clock while allowing deviations. A natural way to minimize deviations from the clock is to minimize the variance of $\tau_{i}/{\hat{b}}_{i}$. This can be achieved by finding *μ* and all *ν_i_* such that *ν_i_* is centered at 1 and $\sum_{i=1}^{2n-2}{\left(\nu_{i}-1\right)}^{2}$ is minimized. Interestingly, the ML function used by LSD (eq. 4) is a weighted version of this approach.

**Fig. 1.**
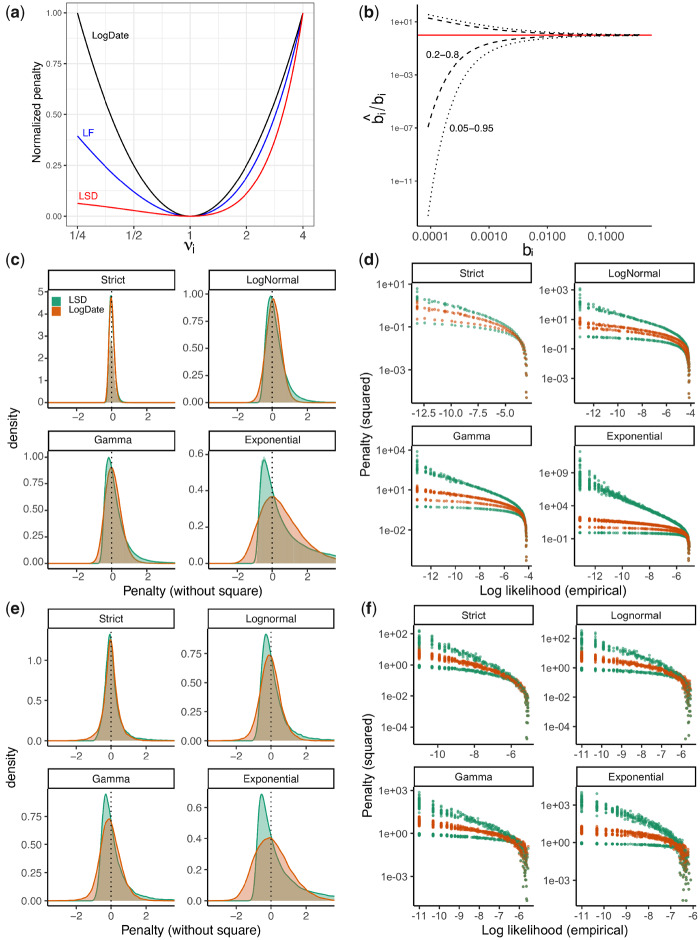
(a) The penalty associated with multiplying a single edge *i* with multiplier *ν_i_* in LSD, LF, and LogDate approaches, as shown in equations (3–5). To allow comparison, we normalize the penalty to be zero at *ν*  =  1 and to be 1 at *ν*  =  4. (*b*) The CI of the ratio between estimated and true branch length using the Poisson model. For this exposition, we assume that the estimated branch length equals the number of substitutions occurring on the branch and follows a Poisson distribution (i.e., JC69 model), divided by sequence length. With these assumptions, the CI for estimate length ${\hat{b}}_{i}$ is between $1/2\chi_{2sb_{i}}^{2}$ and $1/2\chi_{2sb_{i}+2}^{2}$; we draw the CI for α/2=0.05 and α/2=0.2 to get 0.2–0.8 and 0.05–0.095 intervals for $0.0001\leq b_{i}\leq 0.4$. (*c*) and (*e*): Density and histograms of penalty terms (without square) used by LSD ($\mu \tau_{i}/{\hat{b}}_{i}-1$) and LogDate ($\text{log}\;\mu \tau_{i}/{\hat{b}}_{i}$) under different clock models. (*c*) Fixing $\mu \tau_{i}=0.1$, we draw 500,000 rate multipliers (*r_i_*) from LogNormal, Gamma, or Exponential distributions with mean 1 and variance 0.16 for LogNormal and Gamma. For strict clock, *r_i_* = 1. We then draw estimated branch length for each replicate *i* from the Normal distribution with mean $b_{i}=r_{i}\mu \tau_{i}$ and variance $b_{i}/s$ for *s *=* *200. (*e*) The branch lengths are estimated from the sequences using PhyML from simulated sequences of To et al. (2016), as explained in the text. Parameters of rate multiplier distributions match part (*c*). We omit extremely short branches (<0.001) for better visualization. **(***d*) and (*f*) The penalty of LSD and LogDate versus the empirical log-likelihood of estimated length for the models described in (*c*) and (*e*), respectively. To compute the empirical likelihood, we divide estimated branch lengths into small bins and the empirical likelihood of each bin is estimated as the frequency of the data assigned to it. Ideally, increasing likelihood should monotonically decrease the penalty and two points with similar likelihood should have similar penalties. See supplementary figure S2, Supplementary Material online for extended results.

The minimum variance principle results in a fundamental asymmetry: multiplying or dividing the rate of a branch by the same factor are penalized differently (fig 1*a*). For example, the penalty for $\nu_{i}=4$ is more than ten times larger than $\nu_{i}=1/4$. The LF model is more symmetrical than LSD but remains asymmetrical (fig 1*a*). This asymmetry results from the asymmetric distribution of the Poisson distribution around its mean, especially for small mean, in log scale (fig 1*b*). Because of this asymmetry, methods like LSD and LF judge a very small ${\hat{b}}_{i}/b_{i}$ to be within the realm of possible outcomes, and thus penalize $\nu_{i}<1$ multipliers less heavily than $\nu_{i}>1$.

Our method is based on a principle, which we call the *symmetry of ratios*: the penalty for multiplying a branch by a factor of *ν* should be no different than dividing the branch by *ν*. Note that this assertion can only be applicable to true variations of the mutation rate (i.e., ignoring branch length estimation error). We further motivate this principle with more probabilistic arguments below, but here we make the following case. If one considers the distribution of rate *multipliers* for various branches, absent of an explicit model, it is reasonable to assume that compared with an overall rate, branches rates are as likely to increase by a factor of *ν* as they are to decrease by a factor of *ν*. When this statement is true, we shall prefer a method that penalizes *ν* and 1/ν identically. To ensure the symmetry of ratios, we propose taking the logarithm of the multipliers *ν_i_* before minimizing their variance. Minimizing the variance of the rates in log-scale is the essence of our method. It achieves the symmetry, and, as we show below, a better correspondence between penalty and data likelihood.

Log-transformation has long been used to reduce data skewness before applying linear regression (Stynes et al. 1986; Keene 1995; Xiao et al. 2011; Clifford et al. 2013). In molecular dating, it can be argued that log-transformation is implicitly applied in the new version of RelTime (Tamura et al. 2018) where the geometric means between sister lineages replaced the arithmetic means in its predecessor. The improvement in the accuracy of RelTime encourages a wider use of log-transformation in molecular dating. Note that log-transforming the rate multipliers before minimizing their least squares penalty is identical to applying linear least squares after log-transformation of both time and the number of substitutions. In other fields, log-transformation has been used to make the least-squares method more robust to highly skewed distributions (Aban and Meerschaert 2004; Meaney et al. 2007).

#### LogDate Optimization Function

We formulate the LogDate problem as follows. Given $\hat{b}$ and the set of calibration constraints described earlier, we seek to find

(5) $$\begin{array}{cc} x^{*}= & \underset{x}{\arg\min}\sum_{i=1}^{2n-2}{\;\text{log}\;}^{2}(\nu_{i})\;\;\;\text{subject}\;\text{to}\Psi . \end{array}$$

This objective function satisfies the symmetry of ratio property (fig. 1*a*). Since *ν_i_* values are multipliers of rates around *μ*, if we assume *μ* is the mean rate, the LogDate problem is equivalent to minimizing the variance of the log-transformed rate multipliers (around their mean 1). The objective function only depends on *ν_i_*; however, note that *μ* is still included in the constraints and therefore is part of the optimization problem. This setting reduces the complexity of the objective function and speeds up the numerical search for the optimal solution. Since the values of *ν_i_* close to 1 are preferred in equation (5), the optimal solution would push *μ* towards the mean rate.

##### Justification as a Relaxed-Clock Model

After log-transformation, LogDate, similar to LSD, constructs the objective function using the least squares principle (for ease of exposition, here we discuss ordinary least-squares without weights). We can rewrite the objective function of LSD as $\sum_{i}{\left(\mu \tau_{i}/{\hat{b}}_{i}-1\right)}^{2}$ and that of LogDate as $\sum_{i}{\left(\text{log}(\mu \tau_{i}/{\hat{b}}_{i})\right)}^{2}$ and see that both seek to find a global rate *μ* and the time *τ_i_* for each branch to minimize the total deviations of the estimated branches from $\mu \tau_{i}$. This observation may motivate viewing both LSD and LogDate as strict-clock methods. However, the following result justifies viewing LogDate as a relaxed clock method.

We can prove that if the mutation rates *μ_i_* are drawn i.i.d. from a LogNormal distribution with any parameters with mode *μ* and the branches are estimated without error (i.e., ${\hat{b}}_{i}=b_{i}$ for all *i*), then *ν_i_* follows a LogNormal distribution with mode 1 and the LogDate optimization problem is equivalent to finding *ν* values that have maximum joint probability, subject to the constraints. The proof is given in supplementary claim 1, Supplementary Material online.

##### Justification for Symmetry of Ratios

Having shown that LogDate has a justification under the LogNormal distribution, we now compare LogDate and LSD objective functions under a wider range of clock models. Recall that the objective functions of LSD and LogDate are the sum-of-squares of their penalty terms, which are $\mu \tau_{i}/{\hat{b}}_{i}-1$ for LSD and $\text{log}(\mu \tau_{i}/{\hat{b}}_{i})$ for LogDate.

Following the likelihood principle, an ideal objective function must assign equal penalties to data values that are equally likely to occur. Therefore, for an objective function that is written as sum-of-squares of the penalty terms, ideally the probability distribution of its penalty terms (before square) under the model that generates the data should be symmetric around 0 (because of the square). The true distribution of the penalty terms is a function of both clock rate variations and branch length estimation error. Although no objective function can be ideal for all compound models of the rates and the estimation error, a robust objective function should remain close to symmetric and maintain a low skewness under a wide range of models. We now present several theoretical and empirical results comparing LogDate and LSD in terms of the skewness of the distributions of their penalty terms.

First, consider a relaxed clock model of the rates and assume no branch estimation error (i.e., ${\hat{b}}_{i}=\mu_{i}\tau_{i}$). If *μ_i_* follows a LogNormal distribution parameterized by *θ* and *σ* then it is easy to see that $\mu \tau_{i}/{\hat{b}}_{i}=\mu /\mu_{i}$ (penalty of LSD) also follow a LogNormal distribution and the skewness depends on *σ*. In contrast, $\text{log}(\mu \tau_{i}/{\hat{b}}_{i})$ (the penalty of LogDate) follows a Normal distribution, which has skewness 0, and for which least square estimation is the ML estimator. Thus, as stated before, log-transforming is the ML solution if rate multipliers are log-normally distributed.

Now assume *μ_i_* follows a Gamma distribution with mean *μ*. Then $\mu \tau_{i}/{\hat{b}}_{i}=\mu /\mu_{i}$ follows an Inverse Gamma distribution whereas its log-transformation follows a Log-Gamma distribution. We can analytically compute the skewness of the penalty terms of LSD and LogDate and compare them (see supplementary materials for the equations). As shown in supplementary fig. S1, Supplementary Material online, the skewness of LSD is much higher than that of LogDate, especially for higher variance of the gamma rates. Higher skewness of penalty terms violates the likelihood principle mentioned before. Thus, for the two models where we could compute analytical formulas for skewness, we have grounds to prefer LogDate.

Next, we consider the compound impacts of branch length estimation error and rate variation, and we study the question in two ways. One approach is to measure the combined effect of error and true variation by simulating sequence data and measuring ${\hat{b}}_{i}$ for known *b_i_* empirically; here, we use simulations by To et al. (2016) with 1,000 sites and PhyML-inferred trees (details are provided in the Experiments section). The other approach is modeling the compound effect. Although it is hard to know generally how estimated branch length is distributed around its expected value, here, we can follow To et al. (2016) and assume ${\hat{b}}_{i}∼N(b_{i},b_{i}/s)$. The other challenge is that the compound distribution of estimation error and rate multipliers is hard to compute analytically. However, we can easily *generate* a very large number of samples from compound distributions and analyze the empirical distribution to approximate the true distribution.

Inspecting the empirical density of the penalty terms of LSD and LogDate across different clock models result in consistent patterns using both approaches, modeling the compound distribution (fig. 1*c*) and using simulated sequence data (fig. 1*e*). Across three models of rates, Exponential, LogNormal, and Gamma, the distributions of the LogDate penalty terms are always more symmetric than that of LSD. Results are similar for other rate models such as Log-Uniform and are further amplified when the variance is increased (supplementary fig. S2a, Supplementary Material online).

To further explore that relationship between the likelihood and the penalty assigned by LogDate and LSD, we plot the penalty (with square terms) versus the empirical log likelihood of the rate multipliers (fig. 1*d* and *f* and supplementary fig. S2*b*, Supplementary Material online). Ideally, increasing likelihood should monotonically decrease penalty, and points with similar likelihood should have similar penalties. In both modeled and simulated branch lengths and across models, LSD assigns two sets of widely different penalties (one for increased and one for decreased rates) to data with similar likelihood. LogDate, while far from perfect, is much closer to the ideal mapping between likelihood and penalty. Also, for LogNormal with *median* rate multipliers set to 1, we empirically observe a perfectly monotonic relationship between the penalty and likelihood (supplementary fig. S2*b*, Supplementary Material online), as theory suggested.

#### wLogDate Optimization Function

The simple LogDate formulation, however, has a limitation: by allowing rates to vary freely in a multiplicative way, it fails to deal with the varied levels of relative branch error; that is, the ratio of the estimated branch length to the true branch length (${\hat{b}}_{i}/b_{i}$). As ${\hat{b}}_{i}$ is estimated from the sequences, the error of ${\hat{b}}_{i}$ is directly related to the variations in the number of substitutions occurred along the branch *b_i_*. Let us assume sequences follow the Jukes and Cantor (1969) model, and let *N_i_* be the total number of substitutions occurred along branch *i* on a sequence with length *s*. Under Juke-Cantor model, we have $N_{i}∼\text{Poisson}(s\mu \tau_{i})$ and therefore, $\text{var}(N_{i})=s\mu \tau_{i}$. Therefore, the variance of the *expected number of substitutions* around the true branch length is $\text{var}(N_{i}/sb_{i})=s\mu \tau_{i}/s^{2}b_{i}^{2}=1/b_{i}s$. As figure 1*b* shows, when *b_i_* is small, $N_{i}/s$ can easily vary by several orders of magnitude around *b_i_*. Furthermore, the distribution is not symmetric: drawing values several factors smaller than the mean is more likely than drawing values above the mean by the same factor. These analyses predict that the distribution of ${\hat{b}}_{i}/b_{i}$ depends strongly on *b_i_*—with smaller *b_i_* gives higher variance—and is not symmetric.

The variances of the relative error ${\hat{b}}_{i}/b_{i}$ is difficult to compute analytically due to the involvement of the sequence substitution model and the method to estimate ${\hat{b}}_{i}$, which are both unknown. Therefore, we instead use empirical analyses of the estimated branch lengths by PhyML to demonstrate our arguments. Consistent with our prediction, supplementary figure S7*a* and *c*, Supplementary Material online illustrate that the relative error ${\hat{b}}_{i}/b_{i}$ varies more in small branches and the distribution is not symmetric. These properties of the branch length estimates are not modeled in our LogDate formulation and we seek to incorporate them in a refined version of LogDate which will be described below.

Since the true branch length *b_i_* is unknown, a common practice is to use the estimated ${\hat{b}}_{i}$ in place of *b_i_* to estimate its variance as $1/{\hat{b}}_{i}s$. This explains why both LF and LSD objective functions (eqs. 3 and 4) have a weight of ${\hat{b}}_{i}$ for each term of *ν_i_*. Following the same strategy, we propose weighting each ${\;\text{log}\;}^{2}(\nu_{i})$ term in a way that reduces the contribution of short branches to the total penalty, and thus allows more deviations in the log space if the branch is small (and is thus subject to higher error). Since we log-transform *ν_i_* and pursue a model-free approach, explicitly computing the weights to cancel out the variations of relative error among the branches is challenging. However, since the weights should reflect the variance of ${\hat{b}}_{i}/b_{i}$ (logarithmic scale), they should monotonically increase with ${\hat{b}}_{i}$ (fig. 1*b*) to allow more variance for the *relative* errors in short branches than in long branches. We use $\sqrt{{\hat{b}}_{i}}$ as weights, a selection driven by simplicity and empirical performance (shown in the Results section).

The shortest branches require even more care. When the branch is very short, for a limited-size alignment, the evolution produces zero mutations with high probability. For these no-event branches, tree estimation tools report arbitrary small lengths (see supplementary fig. S7, Supplementary Material online), rendering ${\hat{b}}_{i}$ values meaningless for very small branches. To deal with this challenge, the r8s’s implementation of LF Sanderson (2003) collapses all branches with length ${\hat{b}}_{i}<1/s$. To et al. (2016) proposed adding a smoothing constant c/s to each ${\hat{b}}_{i}$ to estimate the variance of ${\hat{b}}_{i}$, where *c* is a parameter that the user can tune. Following a similar strategy, we propose adding a small constant $\tilde{b}$ to each ${\hat{b}}_{i}$. We choose $\tilde{b}$ to be the maximum branch length that produces no substitutions with probability at least 1−α for α∈[0,1]. Recall that *N* is the total number of *actual* substitutions on a branch. Under the Jukes and Cantor (1969) model, it is easy to show that $\arg\max_{\tilde{b}}\mathrm{Pr}(N=0|b=\tilde{b})\geq 1-\alpha =-\frac{1}{s}\text{log}(1-\alpha )$. We choose this value as $\tilde{b}$ and set α=0.01 by default. Thus, we define the wLogDate as follows:

(6) $$\begin{array}{c} x^{*}=\underset{x}{\arg\min}\sum_{i=1}^{2n-1}\sqrt{\tilde{b}+{\hat{b}}_{i}}{\;\text{log}\;}^{2}(\nu_{i})\\ \text{subject}\;\;\text{to}\;\;\Psi . \end{array}$$

#### Solving the Optimization Problem

Both LogDate and wLogDate problems (eqs. 5 and 6) are nonconvex, and hence solving them is nontrivial. The problem is convex if $0\leq \nu_{i}\leq e$. For small clock deviation and small estimation error in ${\hat{b}}_{i}$, the *ν_i_* values should be small so that the problem becomes convex with one local minimum. However, as $\nu_{i}\leq e$ convexity is not guaranteed, we have to rely on gradient-based numerical methods to search for multiple local minima and select the best solution we can find. To search for local minima, we use the SciPy solver with trust-constr Lalee et al. (1998) method. To help the solver work efficiently, we incorporate three techniques that we next describe.

*Computing Jacobian and Hessian matrices* analytically helps speedup the search. By taking the partial derivative of each *ν_i_*, we can compute the Jacobian, *J*, of equation (6). Also, since equation (6) is separable, its Hessian *H* is a (2n−2)×(2n−2) diagonal matrix. Simple derivations give us:

$$\begin{array}{cc} J= & {\left[2\sqrt{\tilde{b}+{\hat{b}}_{1}}\frac{\;\text{log}\;\nu_{1}}{\nu_{1}},\ldots ,2\sqrt{\tilde{b}+{\hat{b}}_{2n-2}}\frac{\;\text{log}\;\nu_{2n-2}}{\nu_{2n-2}}\right]}^{T}\\ \text{and}\;\;\;\;H_{ii}= & 2\sqrt{\tilde{b}+{\hat{b}}_{i}}\frac{1-\text{log}\;\nu_{i}}{\nu_{i}^{2}}\;. \end{array}$$

*Sparse matrix representation* further saves space and computational time. The Hessian matrix is diagonal, allowing us to store only the diagonal elements. In addition, the constraint matrix defined by Ψ is highly sparse. If all sampling times are given at the leaves, the number of nonzero elements in our (n−1)×(2n−1) matrix is O(n log n) (supplementary claim 3, Supplementary Material online). If the tree is either caterpillar or balanced, the number of nonzeroes reduced to Θ(n). Thus, we use sparse matrix representation implemented in the SciPy package. This significantly reduces the running time of LogDate.

*Starting from multiple feasible initial points* is necessary given that our optimization problem is nonconvex. Providing initial points that are feasible (i.e., satisfied the calibration constraints) helps the SciPy solver work efficiently. We designed a heuristic strategy to find multiple initial points given sampling times $t_{1},\ldots ,t_{n}$ of all the leaves (as is common in phylodynamics).

We first describe the process to get a single initial point. We compute the root age *t*_0_ and *μ* using root-to-tip regression (RTT) (Shankarappa et al. 1999). Next, we scale all branches of *T* to conform with Ψ as follow: let $m=\text{arg}{\text{min}}_{i}t_{i}$ (breaking ties arbitrarily). Let *d*(*r*, *i*) denote the distance from the root *r* to node *i* and *P*(*r*, *m*) denote the path from *r* to *m*. For each node *i* in *P*(*r*, *m*), we set $\tau_{i}={\hat{b}}_{i}(t_{m}-t_{0})/d(r,m)$. Then going upward from *m* to *r* following *P*(*m*, *r*), for each edge (*i*, *j*) we compute $t_{j}=t_{i}-\tau_{i}$ and recursively apply the process on the clade *i*. At the root, we set *t_m_* to the second oldest (second minimum) sampling time and repeat the process on a new path until all leaves are processed. Since RTT gives us *μ*, to find *ν* we simply set $\nu_{i}=\mu \tau_{i}/{\hat{b}}_{i}$.

To find multiple initial points, we repeatedly apply RTT to a set of randomly selected clades of *T* and scale each clade using the aforementioned strategy. Specifically, we randomly select a set *S* of some internal nodes in the tree and add the root to *S*. Then, by a postorder traversal, we visit each node u∈S and date the clade under *u* using the scaling strategy described above. We then remove the entire clade *u* from the tree but keep the node *u* as a leaf (note that the age of *u* is already computed) and repeat the process for the next node in *S*. The root will be the last node to be visited. After visiting the root, we have all the *τ_i_* for all *i*. After having all the branches in time unit, we find x to minimize either equation (5) or (6), depending on whether LogDate or wLogDate is chosen. In a tree of *n* leaves, we have $2^{\left(n-1\right)}-1$ ways to select the initial nonempty set *S*, giving us enough room for randomization.

#### Computing Confidence Interval

With the ability of wLogDate to work on any combination of sampling times/calibration points on both leaves and internal nodes (as long as at least two time points are provided), we design a method to estimate the CIs for the estimates of wLogDate. We subsample the sampling times/calibration points given to us repeatedly to create *N* replicate data sets (where *N* is 100 by default, but can be adjusted). Note that our subsampling is not a bootstrapping procedure as node sampling times cannot be resampled with replacement. We then compute the time tree for each replicate to obtain *N* different estimates for the divergence time of each node, from which we can compute their CIs (95% as default). This sampling would work best when we have a fairly large number of calibration points, which is the case in phylodynamic settings where all (or nearly all) sampling times for the leaves are given, or in large phylogenies where abundant calibration points can be obtained from fossils. Although we refer to the resulting intervals as CIs, it is important to recognize that the resampling procedure is not strictly justified via bootstrap theory because subsampling is necessarily without replacement and sampled nodes are not independent of each other.

### Experiments on Simulated Data

#### Phylodynamics Setting

To et al. (2016) simulated a data set of HIV *env* gene. Their time trees were generated based on a birth–death model with periodic sampling times. There are four tree models, namely D995_11_10 (M1), D995_3_25 (M2), D750_11_10 (M3), and D750_3_25 (M4), each of which has 100 replicates for a total of 400 different tree topologies. M1 and M2 simulate intra-host HIV evolution and are ladder-like whereas M3 and M4 simulate inter-host evolution and are balanced. Also, M4 has much higher root-to-tip distance (mean: 57) compared with M1–M3 (22, 33, and 29). Starting from conditions simulated by To et al. (2016), we use the provided time tree to simulate the clock deviations. Using an uncorrelated model of the rates, we draw each rate from one of three different distributions, each of which is centered at the value μ=0.006 as in To et al. (2016). Thus, we set each *μ_i_* to $x_{i}\mu$ where *x_i_* is drawn from one of three distributions: LogNormal (mean : 1.0, std: 0.4), Gamma (α=β=6.05), and Exponential (*λ *= 1). Sequences of length 1,000 were simulated for each of the model conditions using SeqGen Rambaut and Grass (1997) under the same settings as To et al. (2016).

##### Calibrations on Autocorrelated Rate Model

We used the software NELSI and the same protocol as in (Ho et al. 2015) to simulate a data set where the rates are autocorrelated. The data set has 10 replicates each containing 50 taxa. The time trees were generated under birth–death model and the rate heterogeneity through time is modeled by the autocorrelation model (Kishino et al. 2001) with the initial rate set to 0.01 and the autocorrelated parameter set to 0.3. DNA sequences (1,000 bases) were generated under Jukes–Cantor model. We used PhyML (Guindon et al. 2010) to estimate the branch lengths in substitution unit from the simulated sequences while keeping the true topology. These trees are the inputs to wLogDate, RelTime, LF, and DAMBE (Xia 2018) to infer time trees.

### Real Biological Data

#### H1N1 2009 Pandemic

We reanalyze the H1N1 biological data provided by To et al. (2016) which includes 892 H1N1pdm09 sequences collected worldwide between March 13, 2009 and June 9, 2011. We reuse the estimated PhyML (Guindon et al. 2010) trees, 100 bootstrap replicates, and all the results of the dating methods other than wLogDate that are provided by To et al. (2016).

#### San Diego HIV

We study a data set of 926 HIV-1 subtype B *pol* sequences obtained in San Diego between 1996 and 2018 as part of the PIRC study. We use IQTree (Nguyen et al. 2015) to infer a tree under the GTR + Γ model, root the tree on 22 outgroups, then remove the outgroups. Because of the size, we could not run BEAST.

#### West African Ebola Epidemic

We study the data set of Zaire Ebola virus from Africa, which includes 1,610 near-full length genomes sampled between March 17, 2014 and October 24, 2015. The data were collected and analyzed by Dudas et al. (2017) using BEAST and reanalyzed by Volz and Frost (2017) using IQTree to estimate the ML tree and *treedater* to infer node ages. We run LSD, LF, and wLogDate on the IQTree from Volz and Frost (2017) and use the BEAST trees from Dudas et al. (2017), which include 1,000 sampled trees (BEAST-1000) and the Maximum clade credibility tree (BEAST-MCC). To root the IQTree, we search for the rooting position that minimizes the triplet distance (Sand et al. 2013) between the IQTree and the BEAST-MCC tree.

#### Methods Compared

For the phylodynamics data, we compared wLogDate with three other methods: LSD (To et al. 2016), LF (Langley and Fitch 1974), and BEAST (Drummond and Rambaut 2007). For all methods, we fixed the true rooted tree topology and only inferred branch lengths. For LSD, LF, and wLogDate, we used phyML (Guindon et al. 2010) to estimate the branch lengths in substitution units from sequence alignments and used each of them to infer the time tree. LSD was run in the same settings as the QPD* mode described in the original paper (To et al. 2016). LF was run using the implementation in r8s (Sanderson 2003). wLogDate was run with ten feasible starting points. For the Bayesian method BEAST, we also fixed the true rooted tree topology and only inferred node ages. Following To et al. (2016), we ran BEAST using HKY+Γ8 and coalescent with constant population size tree prior. We used two clock models on the rate parameter: the strict-clock (i.e., fixed rate) model and the LogNormal model. For the strict-clock prior, we set clock rate prior to a uniform distribution between 0 and 1. For the LogNormal prior, we set the ucld.mean prior to a uniform distribution between 0 and 1, and ucld.stdev prior to an exponential distribution with parameter 1/3 (default). We always set the length of the MCMC chain to 10^7^ generations, burn-in to 10%, and sampling to every 10^4^ generations (identical to To et al. [2016]).

For the autocorrelated rate model, we compared wLogDate with LF and RelTime (Tamura et al. 2018), which is one of the state-of-the-art model-free dating methods. We randomly chose subsets of the internal nodes (10% on average) as calibration points and created 20 tests for each of the 10 replicates (for a total of 200 tests). We also compared wLogDate with DAMBE using this data set. Because DAMBE can only be run in interactive mode where each calibration point has to be manually placed onto the tree, we could not run DAMBE on the 200 tests with hundreds of calibration points in total. Therefore, we instead ran DAMBE only once on each of the ten trees and infer a unit time tree for each of them (i.e., calibrate the root to be at 1 unit time backward) and compared the results to that of wLogDate. DAMBE does not accept identical sequences so we removed identical sequences from the simulated alignments and trees before running DAMBE and ran wLogDate using these reduced trees to have a fair comparison.

#### Evaluation Criteria

On the simulated phylodynamics data set where the ground truth is known, we compare the *accuracy* of the methods using several metrics. We compute the RMSE of the true and estimated vector of the divergence times (*τs*) and normalize it by tree height. We also rank methods by RMSE rounded to two decimal digits (to avoid different ranks when errors are similar). In addition, we examine the inferred divergence tMRCA and mutation rate. The comparison of methods mostly focuses on point-estimates of these parameters and the accuracy of the estimates (as opposed to their variance). In one analysis, we also compare the CIs produced by wLogDate and BEAST on one model condition (M3 with LogNormal rate distribution). Finally, we examine the correlation between variance of the error in wLogDate and divergence times and branch lengths.

On the simulated data with autocorrelated rate, we show the distributions of the divergence times estimated by wLogDate, LF, and RelTime and report the RMSE normalized by tree height for each replicate. To compare with DAMBE in inferring unit time trees, we report the average relative error of the inferred to the true divergence times. After removing identical sequences, there are 438 internal nodes in total across the 10 tree replicates. For each internal nodes, we compute the relative error of its divergence time inferred by either DAMBE or wLogDate to its true divergence time in the normalized true time tree, which is $|\hat{t_{i}}-t_{i}|/t_{i}$ where $\hat{t_{i}}$ and *t_i_* are the inferred and true divergence times of node *i*, respectively. We report the average relative error per tree replicate and the average of all 438 nodes for DAMBE and wLogDate.

On real data, we show LTT plots (Nee et al. 1994), which trace the number of lineages at any point in time and compare tMRCA times to the values reported in the literature. We also compare the runtime of wLogDate to all other methods in all analyses.

## Results

### Simulated Data for Phylodynamics

We first evaluate the convergence of the ScipPy solver across ten starting points (supplementary fig. S3*a*, Supplementary Material online). LogDate and wLogDate converge to a stable result after 50–200 iterations, depending on the model condition. Convergence seems easier when rates are Gamma or Lognormal and harder when the rates are Exponential. Next, to control for the effect of the starting points on the accuracy of our method, we compare the error of these starting points with the wLogDate optimal point (supplementary fig. S3*b*, Supplementary Material online). In all model conditions, the optimal point shows dramatic improvement in accuracy compared with the starting point. We then compare different weighting strategies for LogDate (supplementary table S4, Supplementary Material online). In all model conditions, the weighting $\sqrt{{\hat{b}}_{i}+\tilde{b}}$, is one of the two best, so it is chosen as the default weighting for wLogDate. Moreover, wLogDate is never worse than LogDate, and under exponential clock models, appropriate weighting results in dramatic improvements (supplementary table S4, Supplementary Material online).

**Fig. 2.**
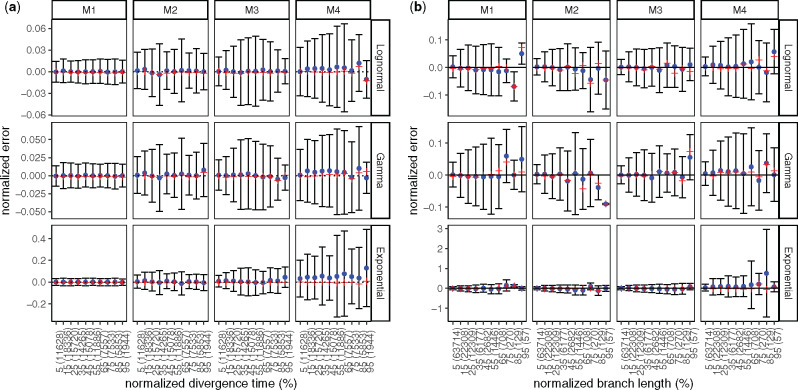
Analyses of wLogDate on inferring branch lengths on simulated data. (*a*) Error normalized by tree height versus divergence time (i.e., the time of the midpoint of each branch); both axes are normalized by the tree height. (*b*) Error versus branch length (in time unit); both axes are normalized by the maximum branch length. For both (*a*) and (*b*), the x-axis is discretized into ten bins of equal size. We label the bins by their median values, relative to either the tree height for (*a*) or the maximum branch length for (*b*). We also show the number of points in each bin in parentheses. Note the small number of points in the final bins in panel (*b*). For each bin, the blue dot represents the mean, the red cross represents the median, and the bar represents one standard deviations around the mean

**Fig. 3.**
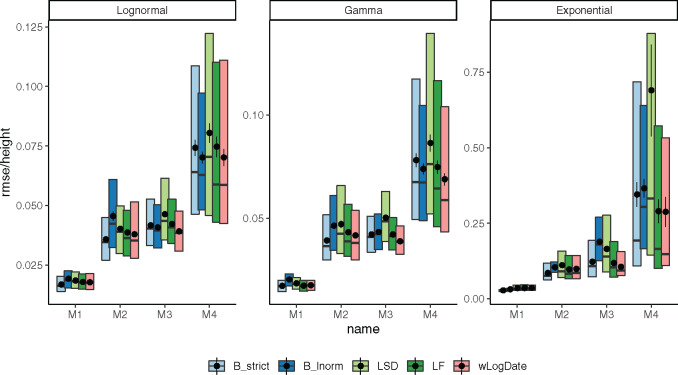
Distributions of RMSE normalized by the tree height for internal node ages inferred by all methods on model trees M1–M4, each with clock models LogNormal, Gamma, and Exponential. Boxes show median, 10% and 90% quantiles; dots and error bars show mean and standard error (100 replicates).

Next, we study the properties of wLogDate estimates in relation to: 1) the age of the node (fig. 2*a*), 2) the length of the true branch in time unit (fig. 2*b*), and 3) the error of the branch lengths (in substitution unit) estimated by PhyML (supplementary fig. S6, Supplementary Material online). Overall, we do not observe a substantial change in the mean estimation error of wLogDate as the node age and the branch length change. The variance, however, can vary with node ages (fig. 2*a*), especially in M3 and M4 model conditions. Moreover, longer branches have a tendency to have higher variance in absolute terms (fig. 2*b*). However, note that the relative error (i.e., log-odds error) dramatically *reduces* as branches become longer (supplementary fig. S6, Supplementary Material online). In studying the effect of the error in branch length estimation, we see that wLogDate can underestimate the branch time if the branch length in substitution unit is extremely underestimated (supplementary fig. S6*a*, Supplementary Material online). In some cases wLogDate underestimates branch times by two order of magnitude or more; all of these cases correspond to super-short branches with substitution unit branch length underestimated by three or four orders of magnitude. As mentioned previously, extremely short estimated branch lengths are often the zero-event branches (supplementary fig. S7, Supplementary Material online), which are unavoidable for short sequences.

We next compare wLogDate with alternative methods, namely LF, LSD, and BEAST with strict-clock and LogNormal clock. Measured by root-mean-square error (RMSE), the accuracy of all methods varies substantially across model trees (M1–M4) and models of rate variation (fig. 3). Comparing methods, for many conditions, wLogDate has the lowest error, and in many others, it is ranked second best (table 1). Across all conditions, wLogDate has a mean rank of 1.75, followed by BEAST with strict clock with a mean rank 2; mean normalized RMSE of wLogDate, LF, BEAST-strict, BEAST-LogNormal, and LSD are 0.072, 0.074, 0.077, 0.087, and 0.116, respectively. In contrast to wLogDate, LSD seems to often underestimate branch times for many short branches even when they are estimated relatively accurately in substitution units (supplementary fig. S6*b*, Supplementary Material online). For all methods, errors are an order of magnitude smaller for the LogNormal and Gamma models of rate variations compared with the Exponential model. In terms of trees, M4, which simulates inter-host evolution and has the largest height, presents the most challenging case for all methods. Interestingly, wLogDate has the best accuracy under all parameters of the M4 tree and also all parameters of M3 (thus, both inter-host conditions). On M1, all methods have very low error and perform similarly (fig. 3).

**Table 1.** Ranking of the Dating Methods under Different Model Conditions.

Model	Clock Model	B_lnorm	B_strict	LF	LSD	wLogDate
M4	LogNormal	**1**	3	4	5	**1**
	Gamma	2	4	3	5	**1**
	Exponential	4	3	2	5	**1**
M3	LogNormal	2	3	3	5	**1**
	Gamma	4	2	2	5	**1**
	Exponential	5	3	2	4	**1**
M2	LogNormal	5	**1**	3	4	2
	Gamma	4	**1**	3	5	2
	Exponential	4	**1**	2	5	3
M1	LogNormal	4	**1**	2	4	2
	Gamma	5	**1**	**1**	4	**1**
	Exponential	2	**1**	3	3	5
Average rank		3.5	2	2.5	4.5	**1.75**

Note.—For each model condition, the average RMSE of all internal node ages is computed and ranked among the dating methods (rounded to two decimal digits). The best method is shown in bold.

Among other methods, results are consistent with the literature. Despite its conceptual similarity to wLogDate, LSD has the worst accuracy. On M1 and M2, LSD is competitive with other methods; however, on M3 and M4, it has a much higher error, especially with the Exponential model of rate variation. With the LogNormal clock model, BEAST-LogNormal is better than BEAST-strict only for M4 but not for M1–M3; in fact, BEAST-LogNormal has the highest error for the M2 condition. This result is surprising given the correct model specification. Nevertheless, BEAST-LogNormal is competitive only under the LogNormal model of rate variation and is one of the two worst methods elsewhere. Thus, BEAST-LogNormal is sensitive to model misspecification. In contrast, BEAST-strict is less sensitive to the model of rate variation and ranks among the top three in most cases. In particular, BEAST-strict is always the best method for intra-host ladder-like trees M1 and M2.

**Fig. 4.**
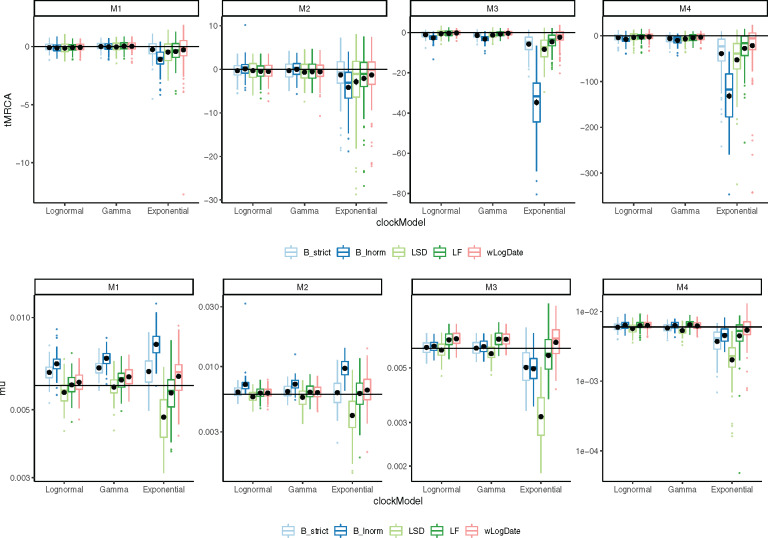
The inferred (top) tMRCA and (bottom) expected mutation rate on different tree models and clock models. Distributions are over 100 replicates. The solid horizontal lines indicate the true mutation rate and tMRCA. Each black dot is the average of the inferred values for each method under each model condition. We remove 6 outlier data points (2 LF, 1 LSD, 2 BEAST-LogNormal, 1 BEAST-Strict) with exceptionally incorrect tMRCA (<−350) in the M4/Exponential model.

Accuracy of time of the Most Recent Common Ancestor (tMRCA) follows similar patterns (fig. 4). Again, the Exponential rate variation model is the most difficult case for all methods, resulting in biased results and highly variable error rates for most methods. In all conditions of M3 and M4, wLogDate has the best accuracy and improves on the second best method by 9–66% (table 2). For M1 and M2, BEAST-strict is often the best method. The mean tMRCA error of wLogDate across all conditions is 4.83 (years), which is substantially better than the second best method, BEAST-strict, with 6.21 (years).

**Table 2.** Mean Absolute Error of the Inferred tMRCA of BEAST_strict, BEAST_lognorm, LF, LSD, RTT, and wLogDate.

Tree	Clock Model	B_strict	B_lnorm	LF	LSD	RTT	wLogDate
M4	LogNormal	6.99	9.50	6.66	7.38	9.28	**6.11** (9%↓)
	Gamma	7.83	10.48	7.02	8.48	8.24	**6.28** (12%↓)
	Exponential	43.5	140.9	116.2	62.2	**31.5**	32.5 (3%↑)
M3	LogNormal	1.37	2.60	1.21	1.39	1.46	**1.03** (17%↓)
	Gamma	1.60	3.14	1.23	1.67	1.42	**0.97** (27%↓)
	Exponential	5.76	34.67	4.87	8.35	3.39	**2.94** (66%↓)
M2	LogNormal	**1.40**	1.41	1.50	1.63	2.19	1.47 (5%↑)
	Gamma	1.54	**1.44**	1.75	1.92	2.56	1.66 (15%↑)
	Exponential	**3.39**	4.59	4.28	5.27	5.23	3.72 (10%↑)
M1	LogNormal	**0.28**	**0.28**	0.30	0.37	0.78	0.30 (7%↑)
	Gamma	**0.27**	0.29	0.32	0.35	0.80	0.30 (11%↑)
	Exponential	**0.60**	1.11	0.79	0.82	1.37	0.69 (15%↑)
Average		6.21	17.54	12.17	8.13	5.68	**4.83**

Note.—For wLogDate, parenthetically, we compare it with the best (↑) or second best (↓) method for each condition. We show percent improvement by wLogDate, as measured by the increase in the error of the second best method (wLogDate or the alternative) divided by the error of the best method. Bold indicates the lowest error for each model condition.

In terms of the mutation rate, the distinction between methods is less pronounced (supplementary table S1, Supplementary Material online). wLogDate is the best method jointly with the two strict clock models BEAST-strict and LF. Overall, even though LF and wLogDate tend to overestimate mutation rates, both have less biased results compared with other methods (fig. 4). LSD and BEAST-LogNormal have the highest errors; depending on the condition, each can overestimate or underestimate the rate but LSD tends to underestimate whereas BEAST-LogNormal tends to overestimate. On M1, wLogDate and LF have a clear advantage over BEAST-strict, which tends to overestimate the rate. On M2, the three methods have similar accuracy. For M3 and M4, BEAST-strict underestimates the rate under the Exponential model of rate variation, and wLogDate and LF are closer to the true value. For all methods, M4 is the most challenging case.

**Fig. 5:**
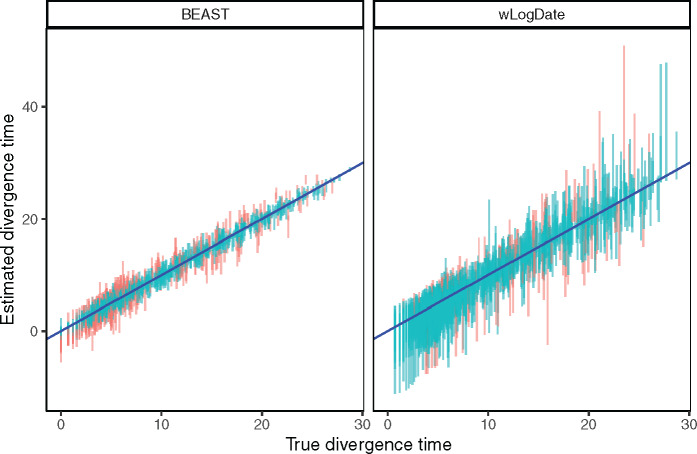
Estimated versus true divergence time. Each bar corresponds to the 95% CI of one node estimate (each of the 109 nodes of the 10 replicates) by BEAST strict clock and wLogDate. Red color is used to mark points where the true time falls outside the CI.

We also compare confidence intervals (CIs) obtained from wLogDate and BEAST (fig. 5). Although wLogDate intervals are on average 2.7 times larger than BEAST, 33% and 12% of the true values fall outside the 95% CI for BEAST and wLogDate, respectively. Thus, whereas both methods underestimate the CI range, wLogDate, with its larger intervals, is closer to capturing the true value in its CI at the desired level.

Finally, we compared all methods in terms of their running time (supplementary table S2, Supplementary Material online). LSD and LF are the fastest methods in all conditions, always taking tens of seconds (less than a minute) on these data. The running time of wLogDate depends on the model condition and can be an order of magnitude higher for Exponential rates than the other two models of rate variation. Nevertheless, wLogDate finishes on average in half a minute to 12 min, depending on the model condition. In contrast, BEAST took close to 1 h with strict clock and close to 2 h with the LogNormal model and even more if run with longer chains; see supplementary table S5, Supplementary Material online.

### Simulated Data with Autocorrelated Rate

**Fig. 6.**
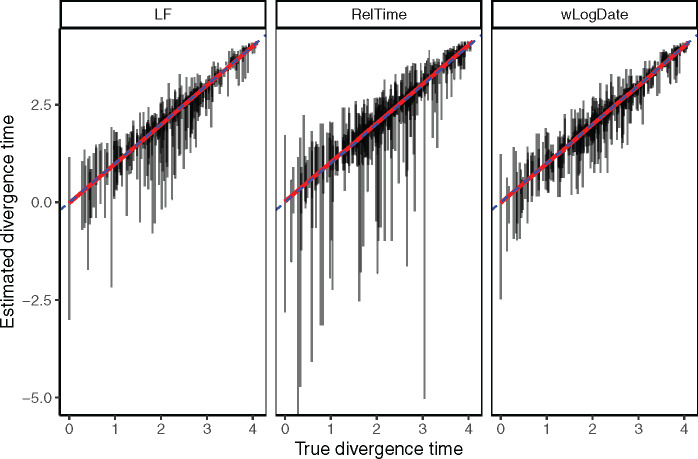
Comparison of LF, RelTime, and wLogDate on the simulated data with autocorrelated rate model. The *y*-axis shows estimated divergence times of uncalibrated internal nodes whereas the *x*-axis shows the true divergence time. Each bar shows the 2.5% and 97.5% quantiles of the estimates of a single node’s divergence time across 20 tests, each of them with different random choices of calibration points (thus, these are not CIs for one run). There are 10 replicate trees, each with 44 uncalibrated nodes (thus, 440 bars in total). This figure discards 2 tests (out of 10 × 20 = 200) where LF produced extremely erroneous time trees (supplementary see fig. S9, Supplementary Material online) for the full results. The RMSE of the uncalibrated internal node ages, normalized by the tree height averaged across all replicates were 0.09, 0.1, and 0.13, respectively, for wLogDate, LF, and RelTime (see supplementary table S7, Supplementary Material online).

In simulations with the autocorrelated rate model, we compare wLogDate with LF and RelTime (fig. 6 and supplementary table S7, Supplementary Material online) and wLogDate to DAMBE (supplementary table S8, Supplementary Material online). The distribution of the estimated divergence time of uncalibrated internal nodes does not show any clear sign of bias in divergence time estimation for either method. All methods seem to give less varied estimates for the younger nodes (i.e., those with higher divergence times) and have more varied estimates for older nodes. In addition, the estimates of wLogDate are more concentrated around the true values than that of LF and RelTime, indicating a better accuracy. In two test cases (out of 200), LF had extremely high error (supplementary fig. S9, Supplementary Material online). Once those two cases are removed, the average RMSE normalized by tree height is 0.09 for wLogDate, 0.10 for LF, and 0.13 for RelTime (supplementary table S7, Supplementary Material online). Comparing with LF and wLogDate, RelTime gives wider distributions of the estimates for a large portion of the nodes. Finally, the comparison in running time of wLogDate and RelTime is shown in supplementary fig. S8, Supplementary Material online.

Comparing with DAMBE in inferring unit time trees, wLogDate has lower error in 6/10 replicates and DAMBE has lower error in the remaining 4 replicates (supplementary table S8, Supplementary Material online). Overall, the average error of wLogDate is 9.40%, which is slightly lower than that of DAMBE at 9.66%.

### Biological Data

**Fig. 7.**
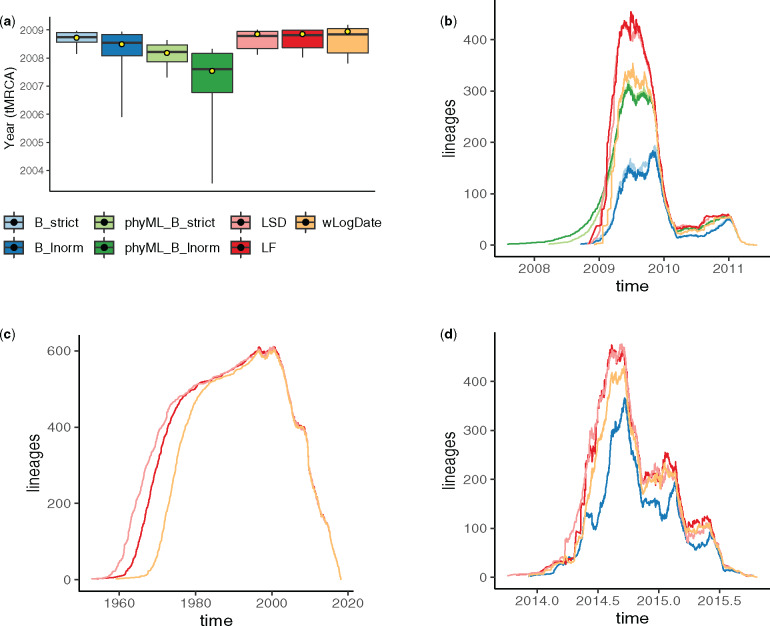
(a) Inferred tMRCA of the H1N1 data set. Boxplots represent the median, maximum, minimum, 97.5% and 2.5% quantiles of the bootstrap estimates for LF, LSD, and wLogDate, and of the posterior distribution for BEAST. Yellow dot shows the inferred tMRCA of the best ML or MAP tree. BEAST was run with four different settings: B_strict and B_lnorm allow BEAST to infer both tree topology and branch lengths, with strict and LogNormal clock models; phyML_B_strict and phyML_B_lnorm fixed the topology to the rooted phyML tree given to BEAST. All other methods (LSD, LF, and wLogDate) were run on the rooted phyML trees. Results for LSD, LF, and BEAST are all obtained from To et al. (2016). (*b*) LTT plot for all methods on the H1N1 data. (*c*) LTT plot of fast methods on the HIV data set. (*d*) LTT plot of BEAST, LSD, LF, and wLogDate on the Ebola data set.

On the H1N1 data set, the best available evidence has suggested a tMRCA between December 2008 and January 2009 (Lemey et al. 2009; Rambaut and Holmes 2009; Hedge et al. 2013). wLogDate inferred the tMRCA to be December 14, 2008 (fig. 7*a*), which is consistent with the literature. LF and LSD both infer a slightly earlier tMRCA (November 10, 2008), followed by BEAST-strict and BEAST-LogNormal (October 2008 and July 2008), and finally BEAST runs using the phyML tree (February 2008 for strict and July 2007 for LogNormal). Although the exact tMRCA is not known on this real data, the results demonstrate that wLogDate, on a real data, produces times that match the presumed ground truth.

On the HIV data set, wLogDate inferred a tMRCA of 1958 with only a handful of lineages coalescing in the 1950s and most others coalescing in 1960s and early 1970s (supplementary fig. S5, Supplementary Material online). The recovered tMRCAs is within the range postulated in the literature for subtype B (Gilbert et al. 2007; Wertheim et al. 2012) and the fact that randomly sampled HIV lineages across US tend to coalesce deep in the tree is a known phenomenon. LF and LSD recovered the tMRCA of 1952 and 1953, respectively. Comparing with wLogDate, these two strict-clock methods postulate an earlier burst of subtype B (fig. 7*c*). We were not able to run BEAST on this data set.

On the Ebola data set, the BEAST-1000 trees obtained from Dudas et al. (2017) inferred the tMRCA to be between September 13, 2013 and January 26, 2014 (95% credible interval) and the BEAST-MCC inferred the tMRCA to be December 5, 2013 as reported by Volz and Frost (2017). Here, wLogDate inferred a tMRCA on December 7, 2013, which is very close to the estimate by BEAST. Both LF and LSD inferred an earlier tMRCA: October 29, 2013 for LF and October 2, 2013 for LSD, but still within the 95% credible interval of BEAST-1000. Lineage-through-time (LTT) plots showed a similar reconstruction by all methods for this data set (fig. 7*d*).

We also compare running times of dating methods on the three real biological data sets (supplementary table S3, Supplementary Material online). LSD was always the fastest, running in just seconds, compared with minutes for LF and wLogDate. LF is faster than wLogDate on the H1N1 and HIV data, whereas on Ebola data, wLogDate is faster. We report the running time for wLogDate as the sequential run of ten independent starting points and note that wLogDate can easily be parallelized. We further tested the scaling of wLogDate with respect to the number of species by subsampling the HIV data set to smaller numbers of species (supplementary fig. S4, Supplementary Material online). The results show that the running time of wLogDate increases slightly worse than quadratically with the incrased number of species.

## Discussion and Future Work

We introduced (w)LogDate, a new method for dating phylogenies based on a nonconvex optimization problem. We showed that by log-transforming the rates before minimizing their variance, we obtain a method that performs much better than LSD, which is a similar method without the log transformation. In phylodynamics settings, our relatively simple method also outperformed other existing methods, including the Bayesian methods, which are much slower. The improvements were most pronounced in terms of the estimation of tMRCA and individual node ages and less so for the mutation rate. Moreover, improvements are most visible under the hardest model conditions, and are also observed when data are generated according to autocorrelated model of rates.

The log transformation results in a nonconvex optimization problem, which is harder to solve than the convex problems solved by LSD and LF. However, we note that the problem is convex for rate multipliers between 0 and *e*. In addition, given the advances in numerical methods for solving nonconvex optimization problems, insistence on convex problems seems unnecessary. Our results indicate that this nonconvex problem can be solved efficiently in the varied settings we tested. The main benefits of the log transformation is that it allows us to define a scoring function that assigns symmetrical penalties for increased or decreased rates (fig. 1*a*); as we argued, this symmetry is a desirable property of the penalty function for several clock models that deviate from a strict clock.

The accuracy of LogDate under varied conditions we tested is remarkable, especially given its lack of reliance on a particular model of rate evolution. We emphasize that the parametric models used in practice are employed for mathematical convenience and not because of a strong biological reason to believe that they capture real variations in rates. Even assuming biological realism of the rate model, the performance of the relaxed clock model used in BEAST was surprisingly low. For example, when rates are drawn from the LogNormal distribution, BEAST-strict often outperformed BEAST-LogNormal, especially in terms of the estimates of tMRCA and the mutation rate. We confirmed that the lower accuracy was not due to the lack of convergence in the MCMC runs. We reran all experiments with longer chains (supplementary table S5, Supplementary Material online) to ensure ESS values are above 300 (supplementary table S6, Supplementary Material online). These much longer runs failed to improve the accuracy of the BEAST-LogNormal substantially and left the ranking of the methods unchanged (supplementary fig. S10, Supplementary Material online).

The LogDate approach can be further improved in several aspects. First, the current formulation of LogDate assumes a rooted phylogenetic tree, whereas most inferred trees are unrooted. Rooting phylogenies is a nontrivial problem and can also be done based on principles of minimizing rate variation (Mai et al. 2017). Similar to LSD, LogDate can be generalized to unrooted trees by rooting the tree on each branch, solving the optimization problem for each root, and choosing the root that minimizes the (w)LogDate objective function. We leave the careful study of such an approach to the future work. Beyond rooting, the future work can explore the possibility of building a specialized solver for LogDate to gain speedup. One approach could be exploiting the special structure of the search space defined by the tree, which is the strategy employed by LSD to solve the least-squares optimization in linear time. Divide-and-conquer may also prove effective. The weighting scheme used in LogDate is chosen heuristically to deal with the deviations of estimated branch lengths from the true branch length. In future, the weighting schema should be studied more carefully, both in terms of theoretical properties and empirical performance.

We described, implemented, and tested LogDate in the condition where calibrations are given as exact times (for any combinations of leaves and internal nodes). While the current settings fit well to phylodynamics data, its application to paleontological data with fossil calibrations is somewhat limited due to the requirements for exact time calibrations (in contrast to the ability to allow minimum or maximum constraints on the ages, or a prior about the distribution of the ages as in BEAST and RelTime). Although the mathematical formulation extends easily, treatment of fossil calibrations and uncertainty of times is a complex topic (Ho and Phillips 2009; Heath 2012) that requires the expansion of this work. Applying LogDate for deep phylogenies would need further tweaks to the algorithm, including changing equality to inequality constraints and the ability to setup feasible starting points for the solver.

In the studies of LogDate accuracy, we have explored various models for rate heterogeinety, including parametric models where rates are drawn independently and identically (i.i.d.) from a distribution (LogNormal, Exponential, and Gamma) and an autocorrelated model where the rates of adjacent branches are correlated. Overall, none of the methods we studied is the best under all conditions. In phylodynamics data, our simulations showed that it is more challenging for all the dating methods to date the phylogenies of the inter-host evolution (M3 and M4) than the intra-host (M1 and M2). wLogDate outperforms other methods for the inter-host phylogenies, regardless of the model of rate heterogeneity. Although all methods have lower error for intra-host trees, BEAST with strict-clock prior is often the best method. However, the differences between BEAST and wLogDate are small and wLogDate is often the second best. Thus, wLogDate works well for virus phylogenies, especially in inter-host conditions. Despite the fact that RelTime explicitly optimizes the rate for each pairs of sister lineages, wLogDate is more accurate than both LF and RelTime on the data where the rates are autocorrelated between adjacent branches. These results show that wLogDate is applicable to a fairly large number of models of the trees and the rates.

Nevertheless, the approach taken by wLogDate suffers from its own limitations. By including a single mean rate around which (wide) variations are allowed, wLogDate is expected to work the best when rates have distribution that are close to being unimodal. However, rates on real phylogenies may have sudden changes leading to bimodal (or multimodal) rate distributions with wide gaps in between modes. For example, certain clades in the tree may have local clocks that are very different from other clades. Such a condition has been studied by Beaulieu et al. (2015) for a data set of seed plants. The authors setup a simulation where there are local clocks on the tree and the mean values of these clocks are different by a factor varying from 3 to 6. Beaulieu et al. (2015) point out that under such condition, especially when the rate shift occurs near the root, BEAST usually overestimates the tMRCA of the Angiosperms (i.e., gives older time) by a factor of 2 (BEAST results from Beaulieu et al. [2015] are reproduced in supplementary fig. S11, Supplementary Material online). We also tested wLogDate, LF, and RelTime on this data set (supplementary fig. S11, Supplementary Material online). In scenario 2 of the simulation, where the rate shift between the two local clocks is extreme (a factor of 6), wLogDate clearly overestimates the age of Angiosperms (by a median of 55%). In this same scenario, RelTime slightly underestimate the age (by 5%). In the other 4 scenarios where the rate shifts are more gentle, wLogDate continue to overestimate the age but by small margins (by 6%, 1%, 2%, and 5%), whereas RelTime underestimates ages also by small margins (3%, 5%, 4%, 3%, and 3%). LF has similar patterns to wLogDate. These results point to a limitation of wLogDate (and the other dating methods) in phylogenies with multiple local clocks.

In addition to multiple clocks, future works should test LogDate under models where rates continuously change with time and have a direction of change. Finally, to facilitate the comparison between different methods, we used the true topology with estimated branch lengths. Future work should also study the impact of the incorrect topology on LogDate and other dating methods.

## Software Availability

The LogDate software is available on https://github.com/uym2/wLogDate (last accessed: September 28, 2020) in open-source format. The command-line python tool is available through PyPI (pip) and conda for easy installation. A link to a web sever making wLogDate available as a web-server is also available from the github page.

## Data Availability

All the data are available on https://github.com/uym2/LogDate-paper (last accessed: September 28, 2020).

## Supplementary Material

Supplementary data are available at *Molecular Biology and Evolution* online.

## Supplementary Material

msaa222_Supplementary_Data
