# Supplementary material for: Log Transformation Improves Dating of Phylogenies
Source: Mol Biol Evol. 2020 Sep 4;38(3):1151–67. doi: 10.1093/molbev/msaa222 (PMC7947844; doi:10.1093/molbev/msaa222)
Supplement: msaa222_Supplementary_Data [file msaa222_supplementary_data.zip › supplementary.pdf]

· doi:10.1093/molbev/sys000 MBE

Supplementary text

Skewness of the penalty terms of LSD and LogDate under Gamma clock model

Suppose the mutation rates μ_i are drawn i.i.d. from a Gamma distribution $\Gamma(\alpha,\beta)$ where α and β are the shape and rate parameters and there is no branch estimation error (i.e. $\hat{b}_i = b_i = \mu_i \tau_i$ for all branch i). Then the mean of μ_i is $\mu = \frac{\alpha}{\beta}$. Define the rate multipliers as $r_i = \frac{\mu_i}{\mu}$, then we have $r_i \sim \Gamma(\alpha,\alpha)$. Recall that the penalty terms of LSD is $\frac{\mu\tau_i}{\hat{b}_i} - 1 = \frac{\mu\tau_i}{\mu_i\tau_i} - 1 = \frac{1}{r_i} - 1$ and the penalty terms of LogDate is $\log r_i$. Note that $\frac{1}{r_i}$ follows an inverse Gamma distribution (with shape α and scale α) and $\log r_i$ a Log-Gamma distribution. Therefore, the skewness of the penalty terms can be computed for LSD to be $\frac{4\sqrt{\alpha-2}}{\alpha-3}$ and for LogDate to be $\frac{\psi^{(3)}(\alpha)}{[\psi^{(2)}(\alpha)]^{3/2}}$ (where $\psi^{(2)}$ and $\psi^{(3)}$ are the digamma and trigamma functions, respectively. Figure S1 shows the skewness of LSD and LogDate when the rate's variance increases.

Algorithm 1 Setup the linear constraints Ψ for tree T given a set of calibration points.

```
function SetupConstraints(T)
    Ψ ← {}
    for w in post-order traversal of T do
        if w is a leaf then
            if w is calibrated then
                nearest_timepoint(w) ← w
            else
                nearest_timepoint(w) ← ∅
        else
            (w1, w2) ← Children(w)
            u ← nearest_timepoint(w1)
            v ← nearest_timepoint(w2)
            if w is calibrated then
                nearest_timepoint(w) ← w
                if u != ∅ then
                    Ψ ← Ψ ∪ ψ(w, u)
                if v != ∅ then
                    Ψ ← Ψ ∪ ψ(w, v)
            else
                if u == ∅ then
                    nearest_timepoint(w) ← v
                else
                    if v == ∅ then
                        nearest_timepoint(w) ← u
                    else
                        nearest_timepoint(w) ← u if (d(w,u) < d(w,v)) else v
                        Ψ ← Ψ ∪ ψ(u, v)
```

Claim 1. *The optimal* $\mathbf{x}^*$ *in Eq. 5 yields a set* $\nu=\{\nu_1,\nu_2,\ldots,\nu_{2n-1}\}$ *that has the maximum joint probability under a model of rate variation where* ν_i *are i.i.d,* $\nu_i \sim LogNormal(\mu_0,\sigma_0^2)$*, and* $Mode(\nu_i)=1$ *for any* μ_0 *and* σ_0^2*, subject to the constraints* Ψ*.*

Proof. We have: $\nu_i \sim LogNormal(\mu_0,\sigma_0^2) \Longrightarrow Mode(\nu_i)=e^{\mu_0-\sigma_0^2} \Longrightarrow 1=e^{\mu_0-\sigma_0^2} \Longrightarrow \mu_0=\sigma_0^2$. In other words, for the conditions with mode 1, we only have one free parameter.

The logarithm of the joint probability of ν under the LogNormal model of rate variation can be written as follows:

$$\begin{aligned} P(\nu_1,\nu_2,\ldots,\nu_{2n-1}|\mu_0,\sigma_0^2) &= \sum_{i=1}^{2n-2}\log\left(\frac{1}{\nu_i\sigma_0\sqrt{2\pi}}\exp\left(-\frac{(\log\nu_i-\mu_0)^2}{2\sigma_0^2}\right)\right) \\ &\propto \sum_{i=1}^{2n-2}\left(-\log\nu_i-\frac{\log^2\nu_i-2\mu_0\log\nu_i}{2\mu_0}\right) \\ &= \sum_{i=1}^{2n-2}-\log^2\nu_i \end{aligned} \tag{S1}$$

Thus, maximizing the joint probability of ν is equivalent to minimizing Eq. 5, subject to the constraints Ψ. □

Lemma 2. *The length of the shortest path from the root of a binary tree to its leaves is at most* $\log n$ *where* n *is the number of leaves in the tree.*

Proof. Consider a rooted binary tree $\mathcal{T}$ with n leaves; let r be the root and h be the length of the shortest path from r to the leaves of $\mathcal{T}$. We need to prove that $h \le \log_2 n$.

Let $\mathcal{D}_i$ be the set of nodes in $\mathcal{T}$ with depth i, that is, $\mathcal{D}_i=\{w\in\mathcal{T}|d(r,w)=i\}$. We first prove that $|\mathcal{D}_i|=2^i\ \forall i\le h$ where $|\mathcal{D}_i|$ denotes the cardinality of $\mathcal{D}_i$. We prove this by induction. The base case $i=0$ holds since the root r is the only node with depth 0. Suppose we have $|\mathcal{D}_k|=2^k$ and $k<h$, we need to prove that if $k+1\le h$ then $|\mathcal{D}_{k+1}|=2^{k+1}$. Note that a node $v\in\mathcal{D}_{k+1}$ if and only if its parent $par(v)\in\mathcal{D}_k$. Because $\mathcal{T}$ is a binary tree, each node in $\mathcal{T}$ must either has no child (leaf node) or two children (internal node). Since $k<h$, there must be no leaf node in $\mathcal{D}_k$, otherwise, a leaf v in $\mathcal{D}_k$ has $d(r,v)=k<h$, which defines a root-to-leaf path that is shorter than h and contradicts the definition of h. Thus, each node in $\mathcal{D}_k$ has exactly 2 children, making $|\mathcal{D}_{k+1}|=2*|\mathcal{D}_k|=2*2^k=2^{k+1}$.

Now we have $|\mathcal{D}_h|=2^h$. To prove that $h\le\log_2 n$, note that $\mathcal{D}_h$ contains a mixture of leaves and internal nodes and each internal node in $\mathcal{D}_h$ must have more than one leaf below it. Therefore, the size of $\mathcal{D}_h$ is at most the size of the leaf set of $\mathcal{T}$; that is, $|\mathcal{D}_h|\le n$. Thus, we have $2^h=|\mathcal{D}_h|\le n \Longrightarrow h\le\log_2 n$. □

Claim 3. *If all the leaves have sampling times and there is no other calibration points given for internal nodes, the matrix corresponding to the constraints* Ψ *setup by Algorithm 1 has* $O(n\log(n))$ *non-zero elements, where* n *is the number of leaves in the input tree* $\mathcal{T}$*.*

Proof. Let $\mathcal{P}(w)$ denote the shortest path from a node w to its leaves and let $|\mathcal{P}(w)|$ denote the length of this path. Let $\mathcal{T}_w$ be the clade of $\mathcal{T}$ below w and let $|w|$ denote the size of this clade (i.e. the number of leaves below w). Applying lemma 2 on $\mathcal{T}_w$, we have $|\mathcal{P}(w)| \leq \log_2|w| \leq \log_2 n$ for all $w \in \mathcal{T}$.

Note that if all leaves have sampling times, Algorithm 1 adds exactly one constraint for each internal node in the tree. For each node w with two children $c_l(w)$ and $c_r(w)$, the non-zero elements of the constraint added when node w is visited must locate on $\mathcal{P}(c_l(w))$, $\mathcal{P}(c_r(w))$, and the two branches $(w,c_l(w))$ and $(w,c_r(w))$. Let η_w denote the number of non-zero elements of the constraint defined by node w, then $\eta_w \leq |\mathcal{P}(c_l(w))| + |\mathcal{P}(c_r(w))| + 1 + 1 \leq 2\log_2 n + 2$. Thus, the total number of non-zeros in all constraints corresponding to the $n-1$ internal nodes is bounded above by $(n-1)(2\log_2 n+2) \in O(n\log n)$.
□

Hybrid rate Angiosperm.

Beaulieu *et al.*((Beaulieu *et al.*, 2015)) simulated a hybrid rate model for a phylogeny of seed plants in which evolutionary rates formed local clocks in certain clades of the tree. The authors simulated that data in 5 scenarios where they change the relative ratios between some clades in the tree, as follow:

- scenario 1 = 3:1 herbaceous to woody
- scenario 2 = 6:1 herbaceous to woody
- scenario 3 = 4:1 angio. to gymno.; 3:1 herbaceous to woody angio.
- scenario 4 = 4:1 angio. to gymno.; 3:1 herbaceous to woody angio.; Gnetales herbaceous angio.
- scenario 5 = 4:1 angio. to gymno.; 3:1 herbaceous to woody angio.; Gnetales woody angio.

The time tree and 100 simulated phylograms for each of these five scenarios were downloaded from the Dryad Repository provided by the authors. We used the provided phylograms to estimate the time tree using wLogDate, RelTime, and LF and compare the estimated age of Angiosperm to the true tree. Without the simulated sequences, we could not run BEAST. However, we show the BEAST results reported by the original study ((Beaulieu *et al.*, 2015)). We aware that the comparison to BEAST must be made with cautions, because the experimental settings were different as we will state below:

- As RelTime cannot run without outgroups, we had to use the 20 species on the clade outside the Angiosperm as outgroups. As such, this entire clade is ignored by RelTime and only 91 species out of 111 are included in the time tree. We used the same setting for wLogDate and LF. Because of this fact, 5 calibration points belong to the 20 species in the outgroups are also discarded out of the total 20 calibration points. However, in their original study, the authors ran BEAST using 20 calibration points (instead of 15 points) to date the full tree with 111 species (instead of 91 species).

- In their original study, the authors gave BEAST a distribution instead of exact-time for each calibration, as opposed to the exact-time points as we used to run LF, RelTime, and wLogDate.

Supplementary figures and tables

Tree Model	Clock Model	BEAST_strict	BEAST_lnorm	LF	LSD	RTT	wLogDate
M1	Lognormal	0.0007	0.0011	**0.0004**	0.0005	0.0007	**0.0004**
	Gamma	0.0009	0.0014	0.0005	**0.0004**	0.0009	0.0006
	Exponential	0.0009	0.0022	**0.0008**	0.0013	0.0013	0.0009
M2	Lognormal	**0.0005**	0.0014	**0.0005**	**0.0005**	0.0008	**0.0005**
	Gamma	**0.0006**	0.0013	0.0007	0.0007	0.0009	0.0007
	Exponential	**0.0013**	0.0038	0.0015	0.0020	0.0019	0.0015
M3	Lognormal	**0.0003**	**0.0003**	0.0006	0.0004	0.0008	0.0006
	Gamma	**0.0003**	**0.0003**	0.0006	0.0004	0.0006	0.0006
	Exponential	0.0010	0.0011	**0.0009**	0.0027	0.0012	**0.0009**
M4	Lognormal	**0.0007**	0.0008	0.0008	**0.0007**	0.0010	0.0008
	Gamma	**0.0006**	0.0007	0.0008	0.0008	0.0010	0.0007
	Exponential	0.0020	**0.0016**	**0.0016**	0.0037	0.0018	0.0017
Average		**0.0008**	0.0013	**0.0008**	0.0012	0.0011	**0.0008**

Table S1. Mean absolute error of the inferred mutation rate of BEAST_strict, BEAST_lognorm, LF, LSD, and wLogDate.

Tree Model	Clock Model	BEAST_strict	BEAST_lnorm	LSD	LF	wLogDate
M1	Lognormal	2784.01	6018.80	15.01	17.47	73.65
	Gamma	2823.09	6082.62	17.81	20.29	83.19
	Exponential	2696.61	5840.18	17.44	19.40	723.61
M2	Lognormal	2425.83	5207.20	18.61	19.94	39.32
	Gamma	2466.24	5303.63	20.12	21.47	81.14
	Exponential	2385.73	5169.07	20.87	22.12	418.01
M3	Lognormal	3848.01	8204.00	35.25	38.07	55.92
	Gamma	3850.71	8211.09	38.71	41.55	65.51
	Exponential	3826.12	6520.19	40.10	43.05	280.55
M4	Lognormal	2914.03	6201.59	28.84	30.40	38.92
	Gamma	2901.59	6184.01	30.78	32.29	41.56
	Exponential	2855.79	6145.03	32.96	34.54	371.97

Table S2. Average running time (seconds) of BEAST_strict, BEAST_lognorm, PhyML + LF, PhyML + LSD, and PhyML + wLogDate with 10 initials on simulated data.

Data	LSD	LF	wLogDate
H1N1 (n=892)	$<$ 1 sec	1 min	3 mins
HIV San Diego (n=904)	$<$ 1 sec	11 mins	24 mins
Ebola (n=1610)	2 secs	4 mins	3 mins

Table S3. Running time of LSD, LF, and wLogDate on biological datasets.

Tree Model	Clock Model	$w_i = 1$	$w_i = \hat{b}_i + \tilde{b}$	$w_i = (\hat{b}_i + \tilde{b})^2$	$w_i = \log(1 + \hat{b}_i + \tilde{b})$	$w_i = \sqrt{\hat{b}_i + \tilde{b}}$	$w_i = \log(1 + \sqrt{\hat{b}_i + \tilde{b}})$
M1	Lognormal	0.020	0.019	0.027	0.019	**0.018**	**0.018**
M1	Gamma	0.020	0.018	0.026	0.018	**0.017**	**0.017**
M1	Exponential	0.363	0.051	0.062	0.048	**0.037**	0.039
M2	Lognormal	0.107	0.043	0.062	0.043	**0.038**	0.042
M2	Gamma	0.100	0.046	0.064	0.046	**0.043**	0.044
M2	Exponential	0.359	0.135	0.166	0.129	**0.099**	0.100
M3	Lognormal	**0.039**	0.051	0.134	0.050	**0.039**	**0.039**
M3	Gamma	0.041	0.050	0.132	0.049	**0.039**	**0.039**
M3	Exponential	0.220	0.173	0.349	0.168	0.105	**0.103**
M4	Lognormal	0.074	0.098	0.172	0.085	**0.070**	0.071
M4	Gamma	0.098	0.086	0.172	0.082	0.070	**0.069**
M4	Exponential	0.578	0.397	1.042	0.349	0.301	**0.283**

Table S4. Average RMSE of the internal node ages inferred by different weight functions for LogDate. Numbers are rounded to the closest 3 decimal digits. Recall that $\hat{b}_i$ is the estimated branch length and $\tilde{b}$ is a small constant.

Tree Model	MCMC Chain	Relative Error	Run Time (hours)
M1	10^7	1.00	1.66
M2	5×10^7	0.99	7.6
M3	5×10^7	0.99	11.3
M4	20×10^7	0.98	35.6

Table S5. BEAST convergence analysis: BEAST was run on Lognormal clock models with the correct prior. For each tree model, we run BEAST with a sufficiently long MCMC chain to ensure the effictive-sample-size (ESS) of all parameters are at least 200. We report the length of the MCMC chain, relative error of node age estimates with respect to BEAST using 10 millions MCMC chain, and the running time.

Tree Model	Posterior ESS	Likelihood ESS	MeanRate ESS	RootHeight ESS
M1	420.5	753.3	382.0	406.9
M2	766.9	4224.4	430.1	647.3
M3	1242.3	4024.1	531.0	430.3
M4	1254.9	16336.7	744.1	847.2

Table S6. BEAST convergence analysis: BEAST was run on Lognormal clock models with the correct prior. For each tree model, we run BEAST with a sufficiently long MCMC chain to ensure the effictive-sample-size (ESS) of all parameters are at least 200. We report the average ESS of posterior, likelihood, rootHeight, and meanRate of the first 10 replicates of each tree model.

Replicate	LF	RelTime	wLogDate
1	**0.10**	0.26	0.11
2	0.12	0.21	**0.10**
3	0.11	**0.09**	0.10
4	**0.08**	0.10	0.22
5	**0.05**	0.26	0.06
6	0.10	**0.06**	0.08
7	0.09	0.10	**0.08**
8	0.07	**0.06**	**0.06**
9	0.09	0.08	**0.07**
10	0.17	0.08	**0.07**
Average	0.10	0.13	**0.09**

Table S7. Comparison of LF, RelTime, and wLogDate on autocorrelated rate dataset. The Root-mean-square error (RMSE) of un-calibrated internal node ages is normalized by the tree height and reported for each replicate. Results discarded the two tests where LF produced extremely erroneous time tree. Refer to Fig. S9 for a complete picture.

Replicate	DAMBE	wLogDate
1	**7.69**	11.54
2	13.25	**10.88**
3	9.04	**8.30**
4	9.37	**9.10**
5	**3.78**	4.09
6	**4.86**	4.92
7	13.98	**12.46**
8	6.91	**6.43**
9	13.36	**10.93**
10	**14.81**	15.88
**Average**	9.66	**9.40**

Table S8. Average relative error (%) of DAMBE and wLogDate in estimating unit time trees on the autocorrelated rate model. For each of the 438 internal nodes across the 10 simulated trees, the relative errors of the inferred divergence times by DAMBE and wLogDate to that of the true normalized time tree are computed. The average error of all nodes per tree replicate and the average error of all 438 nodes are shown for each method.

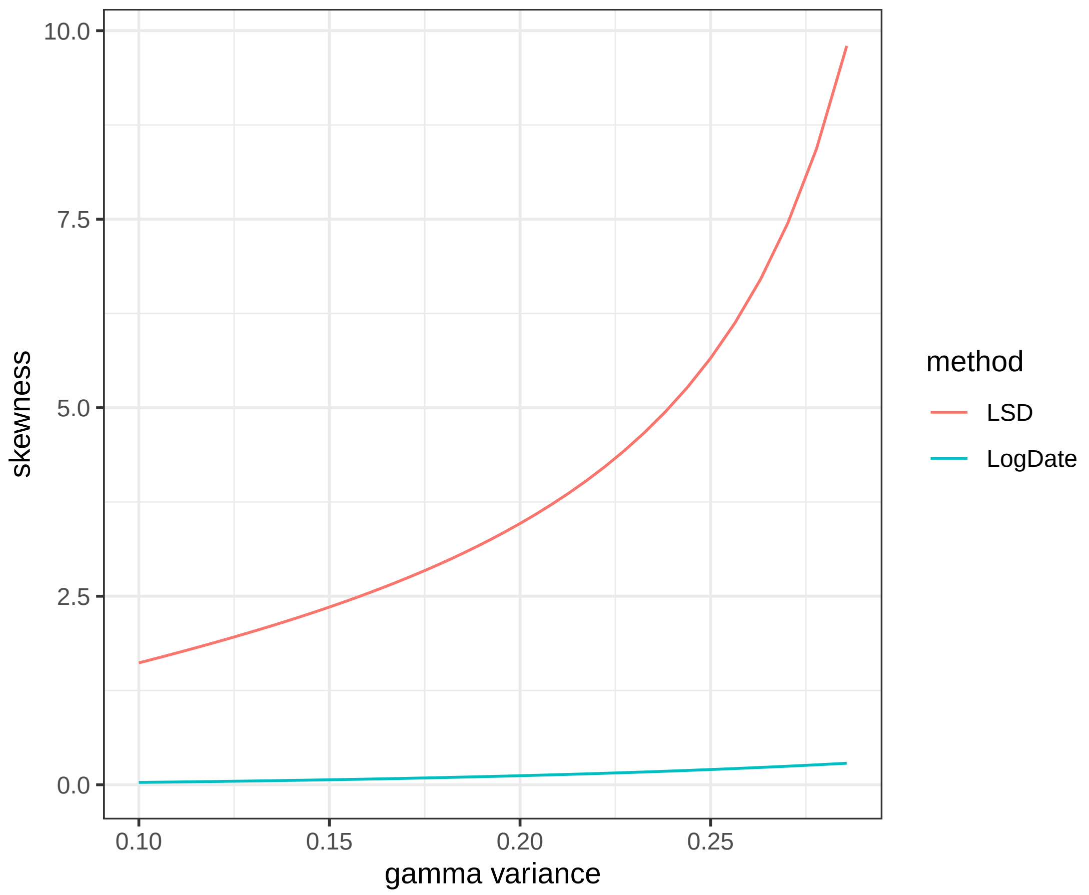

FIG. S1. The skewness of the LSD and LogDate penalty terms when the rate multipliers r_i are drawn i.i.d. from a Gamma distribution with different α. The x-axis shows the variance of r_i and the y-axis shows the skewness of the penalty terms of LSD and LogDate.

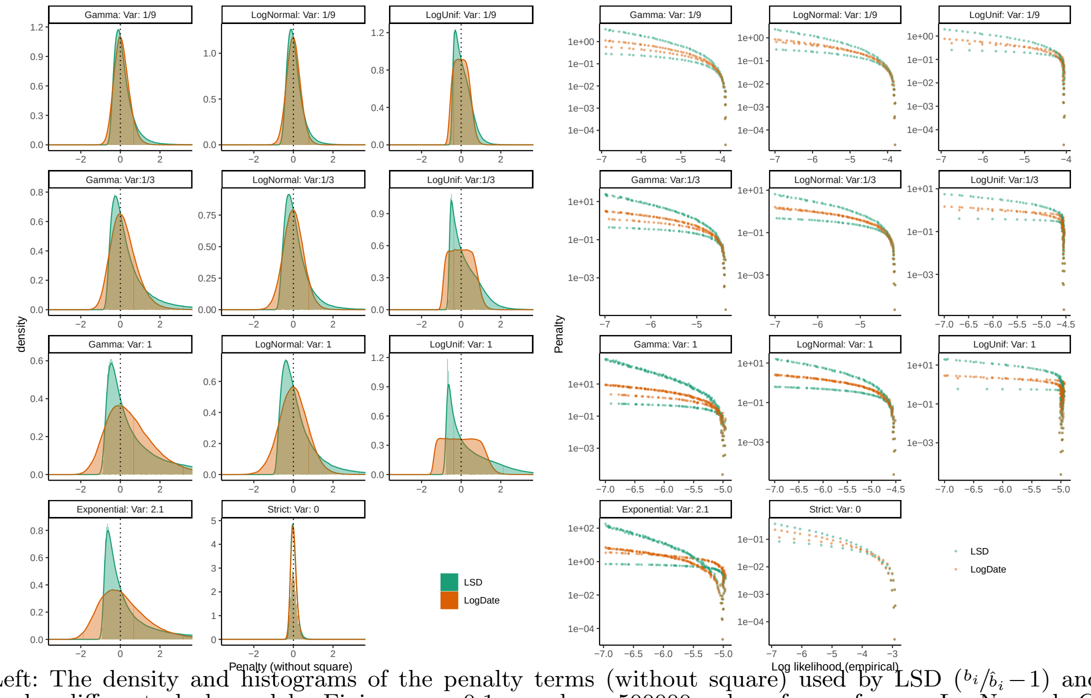

FIG. S2. Left: The density and histograms of the penalty terms (without square) used by LSD ($b_i/\hat{b}_i - 1$) and LogDate ($\log b_i/\hat{b}_i$) under different clock models. Fixing $\mu\tau_i = 0.1$, we draw 500000 values for r_i from a LogNormal, Gamma, or Exponential distribution with median equal to 1 and variance equal to 1/9, 1/3, 1, or 2.1. To simulate strict clock, we fixed $r_i = 1$. We then simulate estimated branch length for each replicate following the To *et al.* (2015) model, by drawing $\hat{b}_i$ from a normal distribution with mean $b_i = r_i \mu \tau_i$ and variance b_i/s. Right: The penalty of LSD and LogDate versus the empirical log-likelihood of $\hat{b}_i$ for the models described above. To compute the empirical likelihood, we divide $\hat{b}$ observations into small bins and the empirical likelihood of each bin is estimated as the frequency of the data assigned to it. Ideally, increasing likelihood should monotonically decrease penalty. LogDate is closer to this idea than LSD across all models, especially with higher variance of r_i.

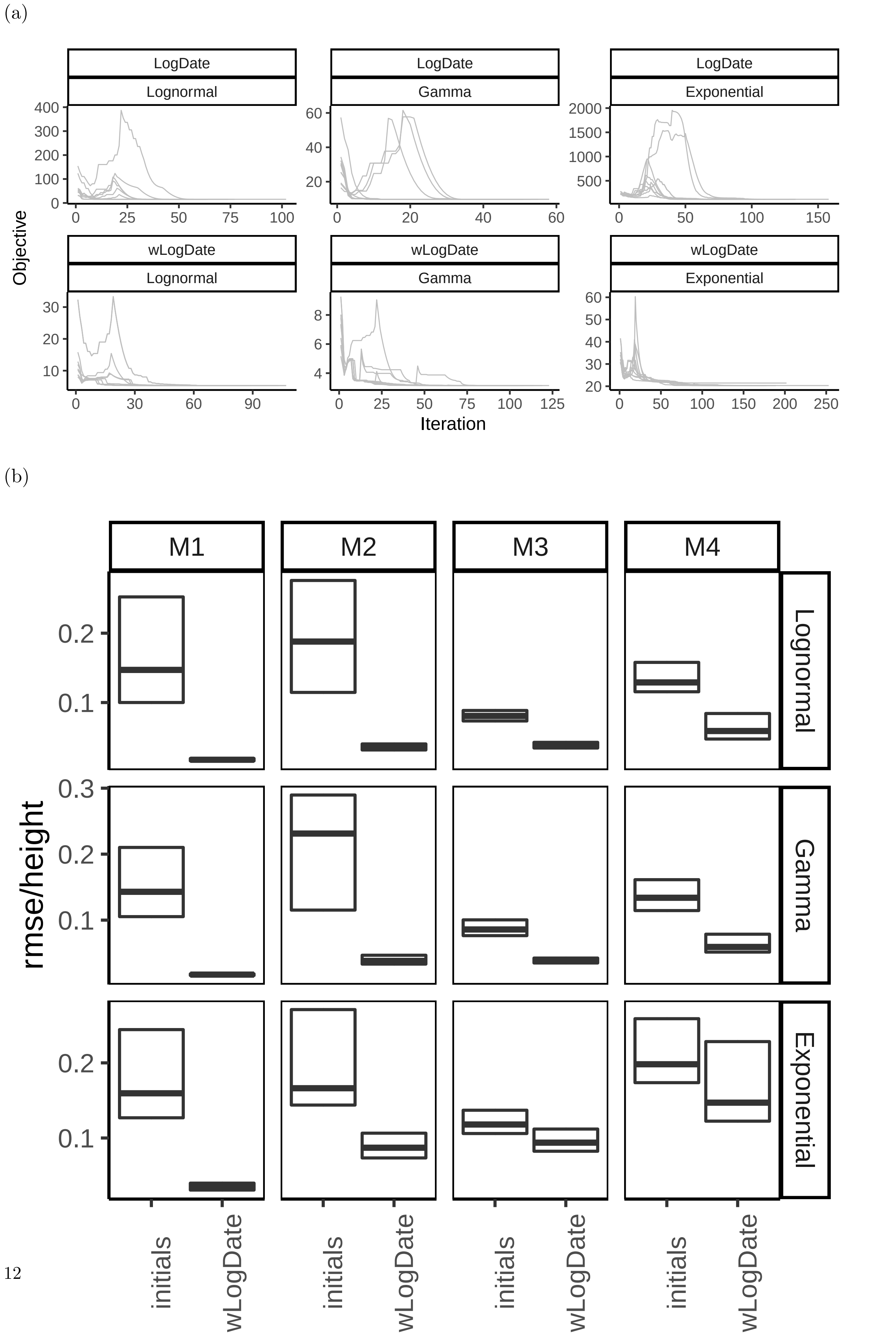

FIG. S3. (a) Objective value versus iteration of the LogDate and wLogDate runs on one arbitrarily selected simulated tree (M4, replicate 2). Each of the two methods were run using 10 random initial points generated using the strategy described in the main text. (b) Normalized root-mean-square error of wLogDate versus the 10 initials used to run wLogDate.

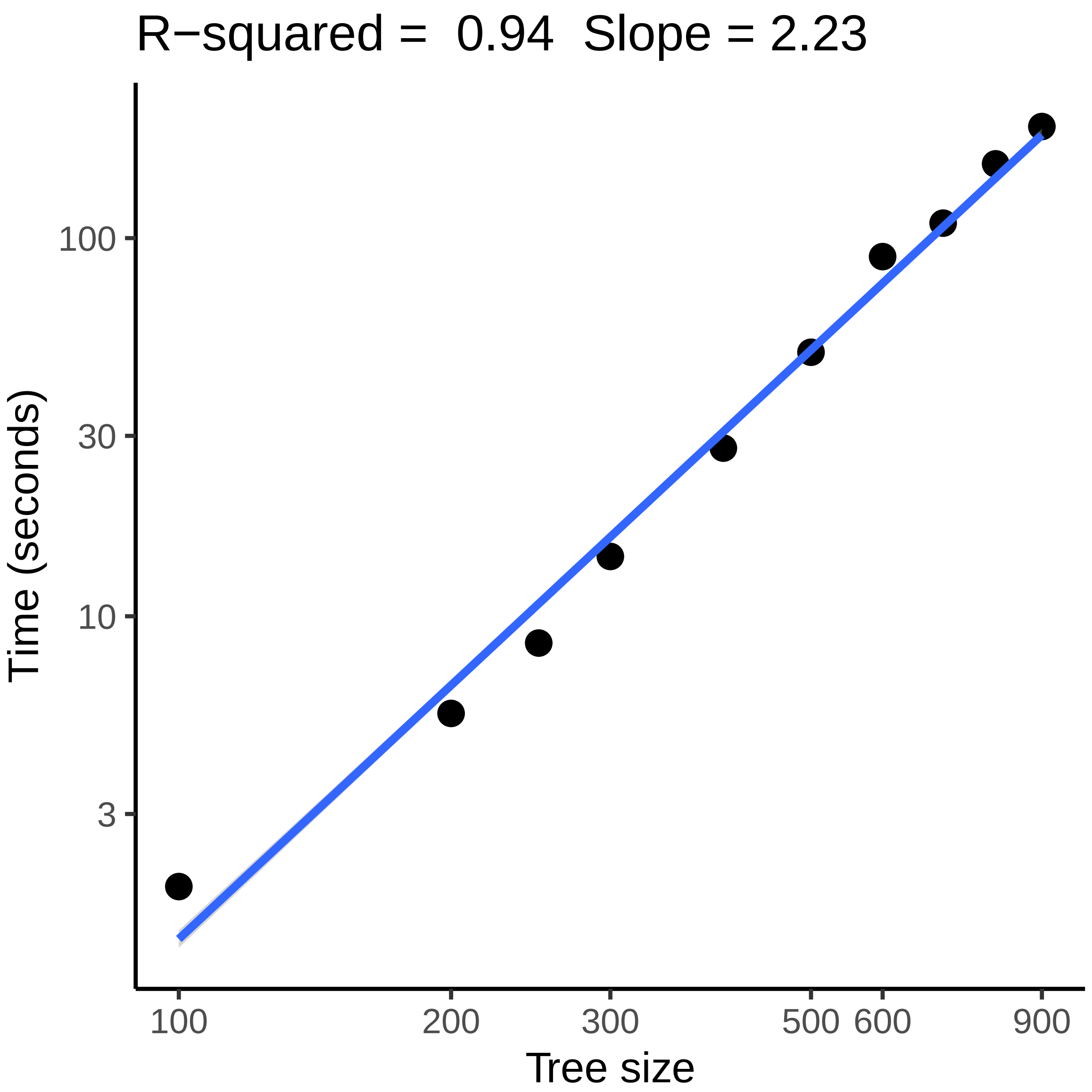

FIG. S4. Running time of wLogDate on random subsets of the HIV dataset. For each tree size, wLogDate was run 100 times on 10 random subsets each with 10 initial points. Each dot represents the average run time of wLogDate per subset per initial point. Both axes are scaled in log (base 10). The slope of the line (2.23) shows the polynomial degree of the running time increase of wLogDate. Thus, wLogDate scales slightly worse than quadratically with increased numbers of species.

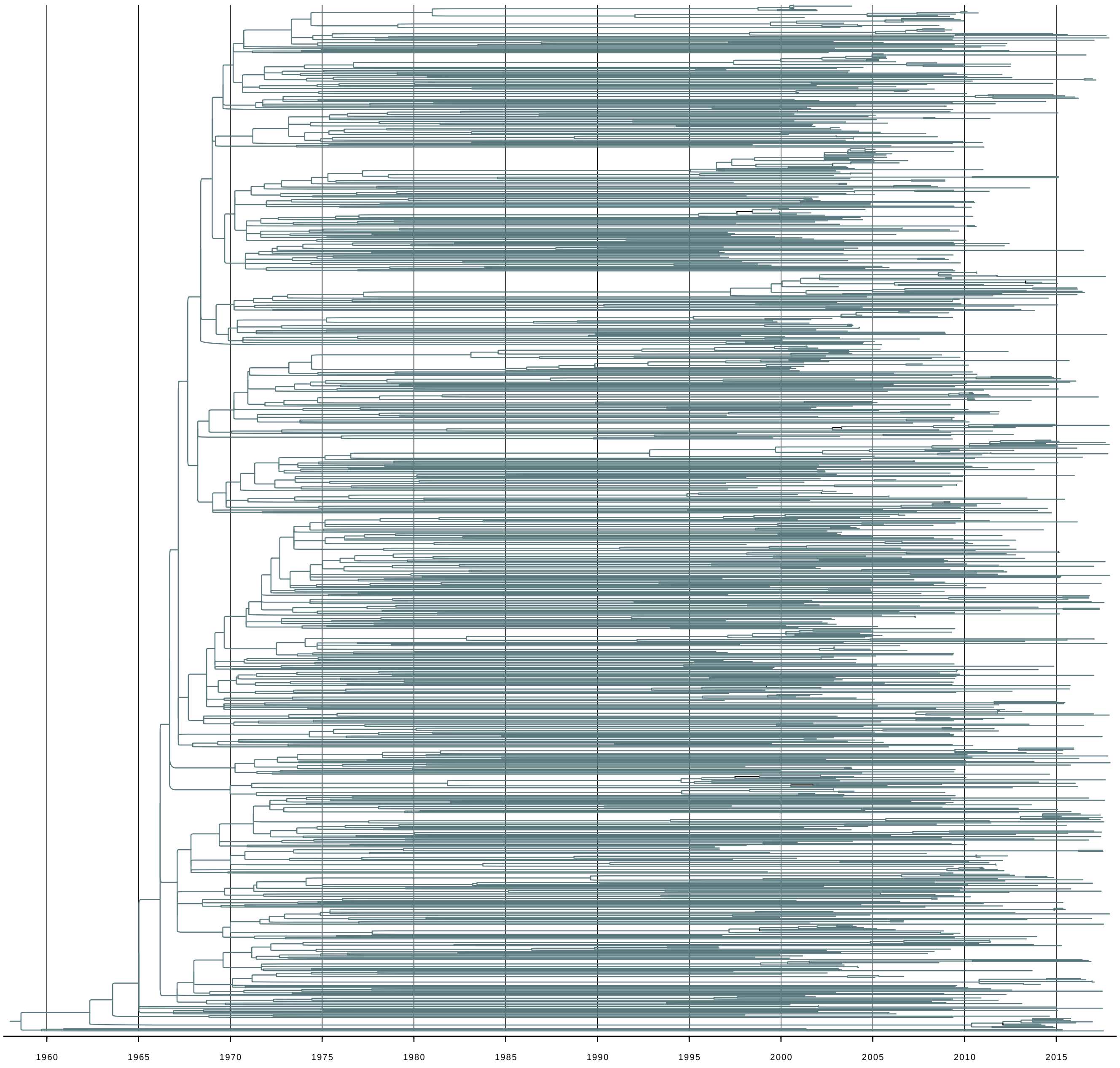

FIG. S5. A TimeTree of San Diego HIV epidemic according to wLogDate.

(a) wLogDate

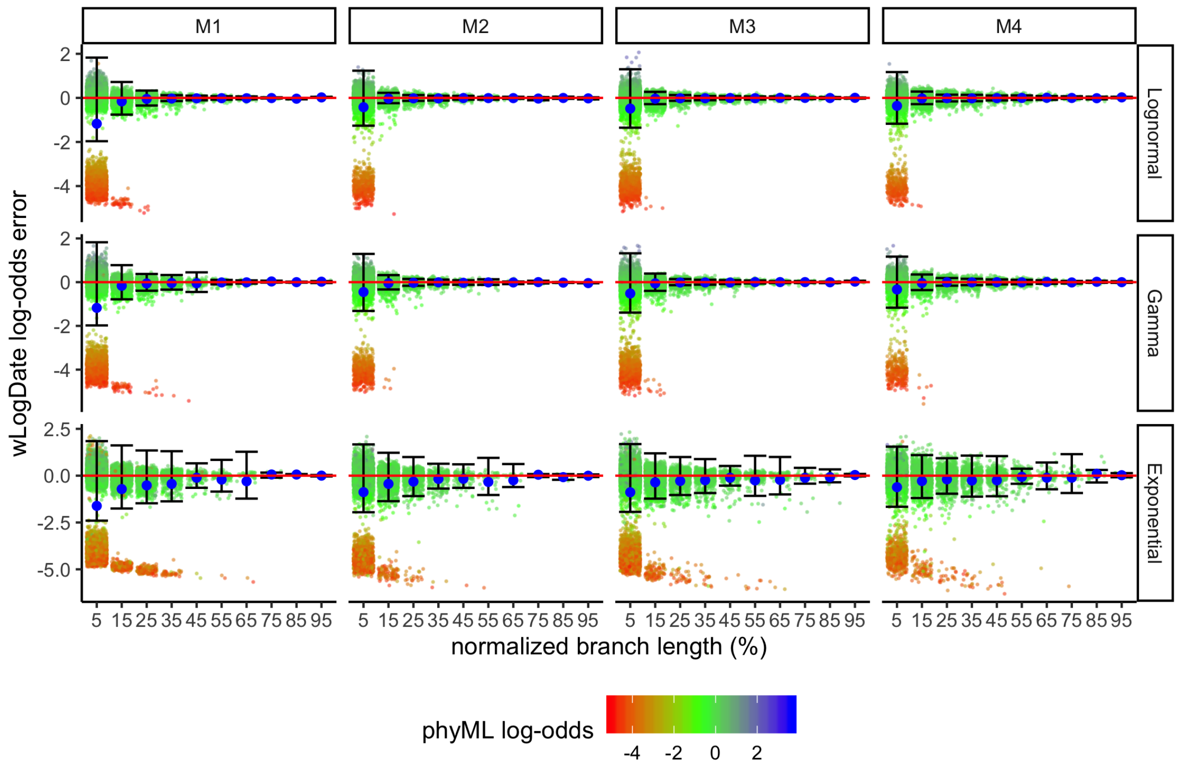

(b) LSD

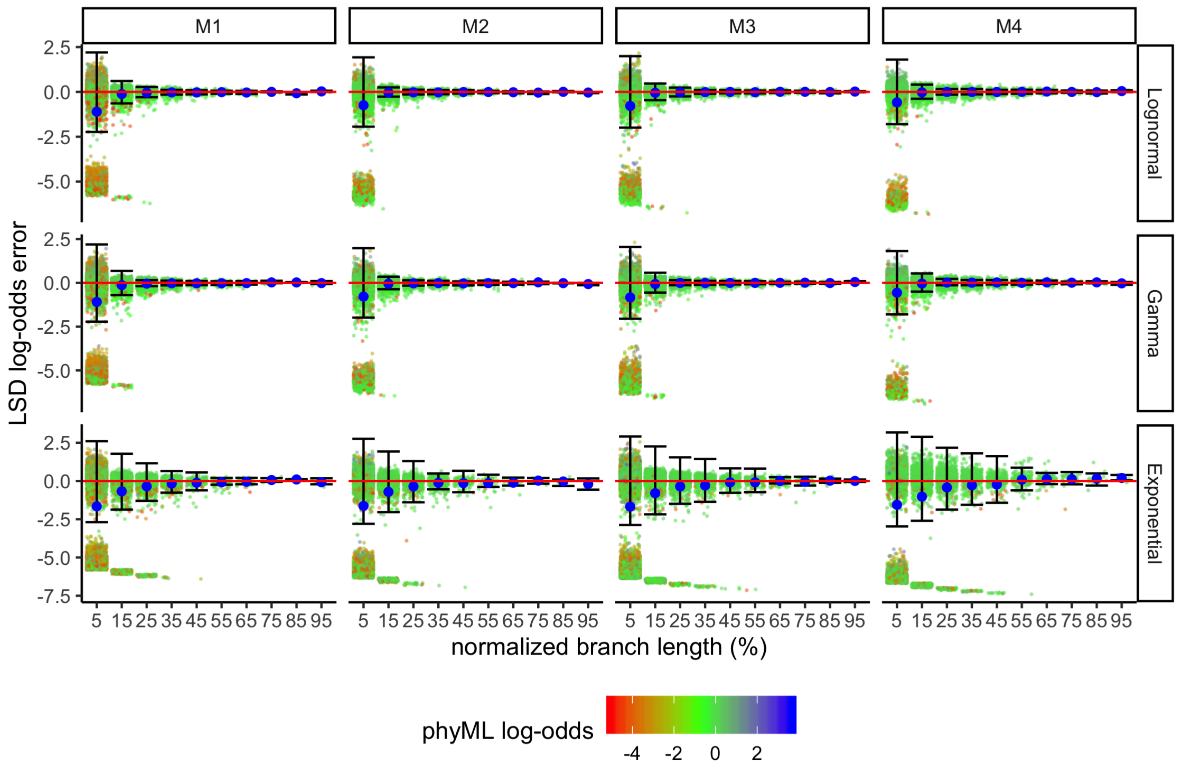

FIG. S6. Effects of PhyML estimation error on wLogDate and LSD performance. Figure shows log-odds error of (a) wLogDate and (b) LSD versus true branch length (in time unit); x-axis is normalized by the maximum tree branch; dots are colored by log-odds error of phyML estimates; large blue dots show means and bars show one standard deviations around medians.

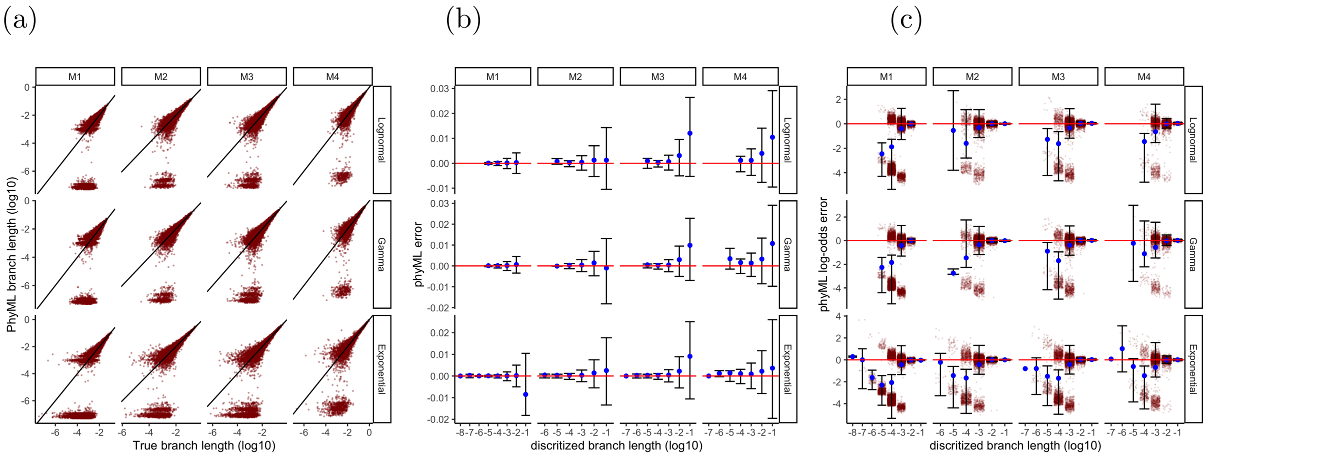

FIG. S7. Analyses of the estimated branch lengths using PhyML on simulated data. (a) Estimated versus true branch lengths (expected number of substitutions per site); axes scaled in log10. (b) Error versus true branch lengths; blue dots represent means and bars represent standard deviations around medians. (c) Log-odds error versus true branch lengths; blue dots represent means and bars represent standard deviations around medians.

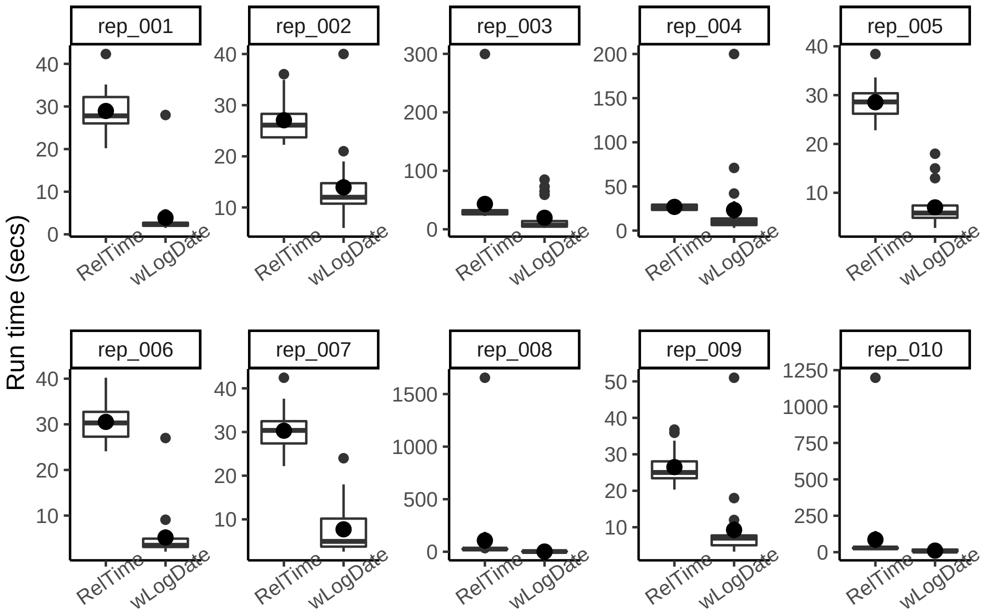

FIG. S8. Run time of wLogDate and RelTime on the 10 replicates. Box plots show distributions for the 20 tests.

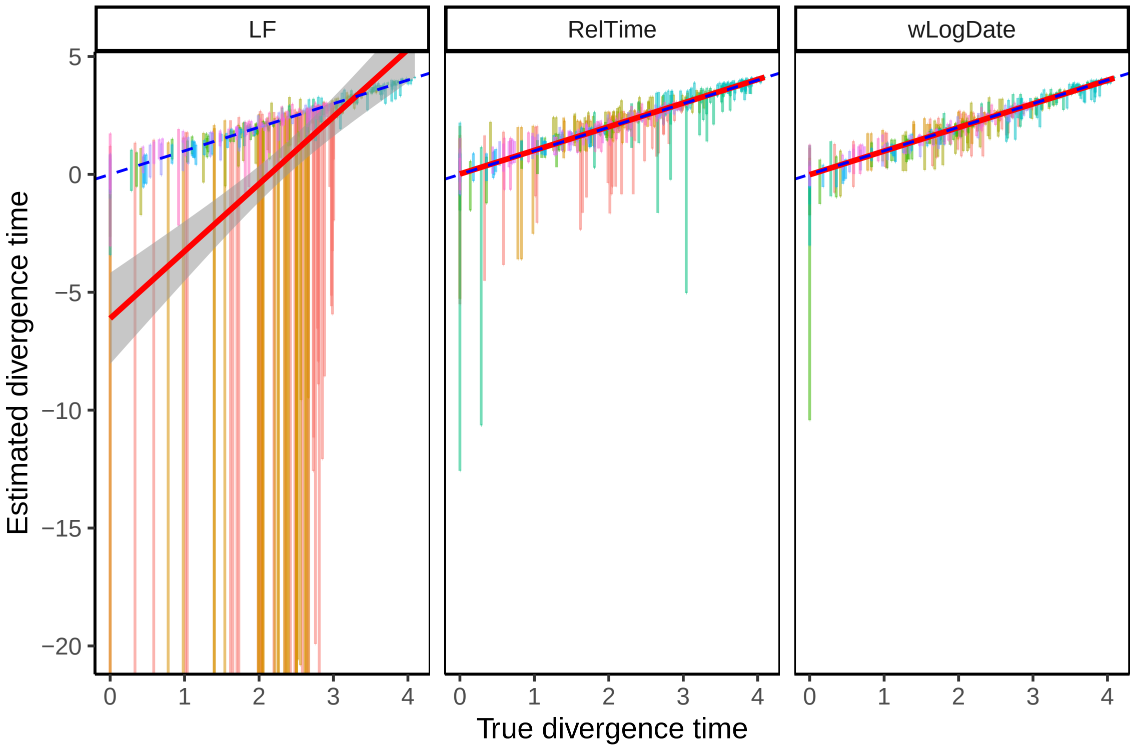

FIG. S9. Comparison of LF, RelTime, and wLogDate on the simulated data with autocorrelated rate model. The y-axis shows estimated divergence times of uncalibrated internal nodes while the x-axis shows the true divergence time. Each bar shows the 2.5% and 97.5% quantiles of the estimates of a single node's divergence time across 20 tests, each of them with different random choices of calibration points (thus, these are not CIs for one run). There are 10 replicate trees, each with 44 uncalibrated nodes (thus, 440 bars in total). There are two tests where LF produces extremely erroneous time trees (test 2 of replicate 1 and test 16 of replicate 2) and were discarded in Fig. 6 in the main text. The normalized RMSE of LF are 41.3 and 167.8 for these two tests, while the overall error without these two tests is 0.09. Here we show the full results for completeness. Colors are used to distinguish between replicates.

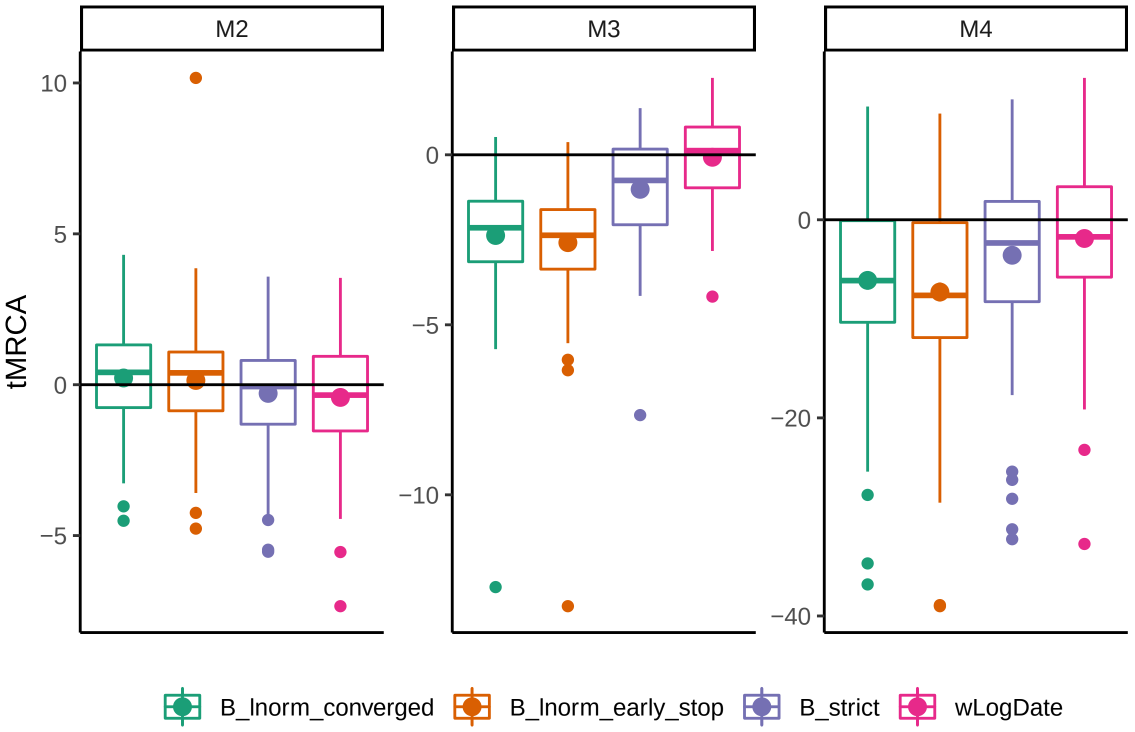

FIG. S10. Estimation of the tMRCA of $M2$, $M3$, and $M4$ of the simulated data with Lognormal clock model. For each model, BEAST was run with 3 conditions: B_strict uses the strict-clock prior, B_lnorm_early_stop uses Lognormal clock prior with MCMC chain of 10 millions, and B_lnorm_converged uses Lognormal clock prior with elongated MCMC chain to guarantee convergence (refer to table S6 for parameters and convergence check.)

References

Beaulieu, J. M., O'Meara, B. C., Crane, P., and Donoghue, M. J. 2015. Heterogeneous Rates of Molecular Evolution and Diversification Could Explain the Triassic Age Estimate for Angiosperms. *Systematic Biology*, 64(5): 869–878.

To, T.-H., Jung, M., Lycett, S., and Gascuel, O. 2015. Fast Dating Using Least-Squares Criteria and Algorithms. *Systematic Biology*, 65(1): 82–97.

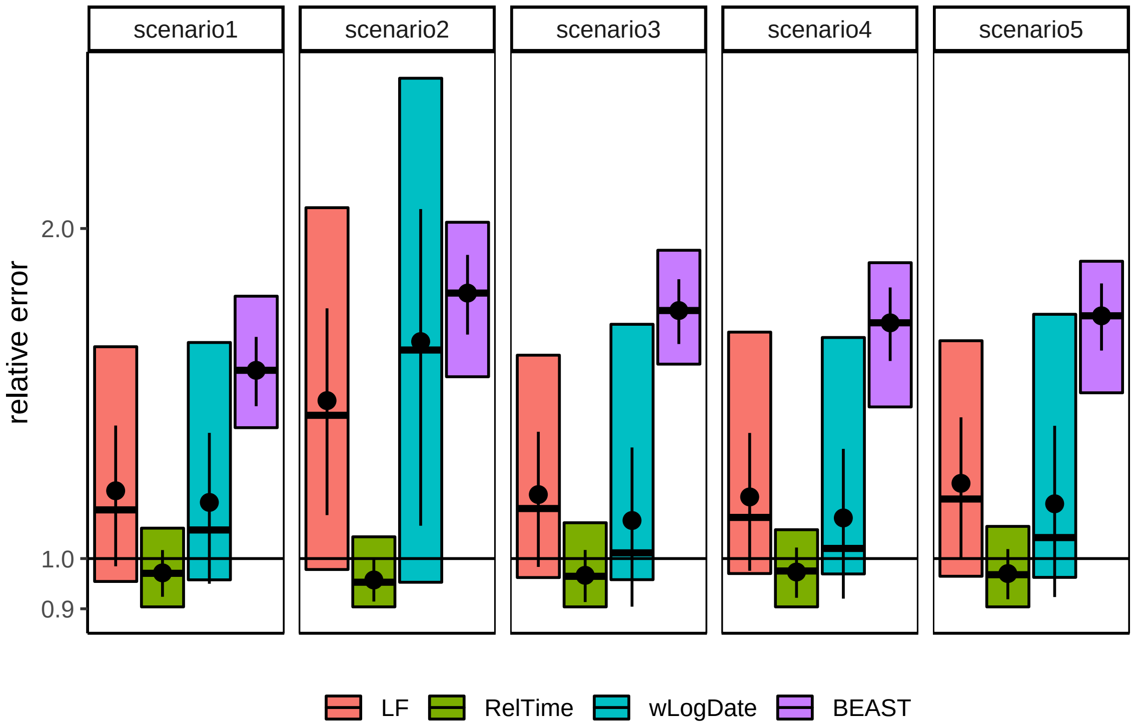

FIG. S11. Relative error of wLogDate, RelTime, and LF on inferring the Angiosperm's age on different settings of the simulation by (Beaulieu *et al.*, 2015). Boxplots show median with 95% CI. Point ranges show mean with one standard deviation. Results for BEAST was obtained from the original study (Beaulieu *et al.*, 2015) on the same dataset but some different settings for the calibrations.
